# The MICOS Complex Regulates Mitochondrial Structure and Oxidative Stress During Age‐Dependent Structural Deficits in the Kidney

**DOI:** 10.1111/acel.70534

**Published:** 2026-05-11

**Authors:** Prasanna Katti, Praveena Prasad, Sepiso K. Masenga, Prasanna Venkhatesh, Zer Vue, Andrea G. Marshall, Benjamin Rodriguez, Han Le, Edgar Garza‐Lopez, Alexandria Murphy, Brenita Jenkins, Ashlesha Kadam, Jianqiang Shao, Amber Crabtree, Pamela Martin, Chantell Evans, Mark A. Phillips, David Hubert, Nelson Wandira, Okwute M. Ochayi, Dhanendra Tomar, Clintoria R. Williams, Jennifer Gaddy, Briar Tomeau, LaCara Bell, Taneisha Gillyard, Markis' Hamilton, Vineeta Sharma, Mohd Mabood Khan, Elma Zaganjor, Olujimi A. Ajijola, Estevão Scudese, Tyne W. Miller Fleming, André Kinder, Chandravanu Dash, Anita M. Quintana, Bret C. Mobley, Julia D. Berry, Pooja Jadiya, Dao‐Fu Dai, Annet Kirabo, Oleg Kovtun, Jenny C. Schafer, Sean Schaffer, Renata Oliveira Pereira, Debra D. Murray, Joyonna Gamble‐George, Melanie R. McReynolds, Antentor Hinton

**Affiliations:** ^1^ Department of Biology Indian Institute of Science Education and Research (IISER) Tirupati Andhra Pradesh India; ^2^ Department of Biochemistry and Molecular Biology The Huck Institute of the Life Sciences, Pennsylvania State University University Park Pennsylvania USA; ^3^ Department of Molecular Physiology and Biophysics Vanderbilt University Nashville Tennessee USA; ^4^ Department of Physiological Sciences, Pathology and Microbiology, HAND Research Group School of Medicine and Health Sciences Livingstone Zambia; ^5^ Department of Internal Medicine University of Iowa Iowa City Iowa USA; ^6^ Department of Internal Medicine, Section of Cardiovascular Medicine Wake Forest University School of Medicine Winston‐Salem North Carolina USA; ^7^ Central Microscopy Research Facility, University of Iowa Iowa City Iowa USA; ^8^ Department of Biomedical Sciences School of Graduate Studies, Meharry Medical College Nashville Tennessee USA; ^9^ Department of Cell Biology Duke University School of Medicine Durham North Carolina USA; ^10^ Department of Integrative Biology Oregon State University Corvallis Oregon USA; ^11^ Department of Medicine Vanderbilt University Medical Center Nashville Tennessee USA; ^12^ Department of Human Physiology Baze University Abuja Nigeria; ^13^ Department of Neuroscience, Cell Biology and Physiology Wright State University Dayton Ohio USA; ^14^ Department of Biochemistry, Cancer Biology, Neuroscience and Pharmacology Meharry Medical College Nashville Tennessee USA; ^15^ UCLA Cardiac Arrhythmia Center, University of California Los Angeles California USA; ^16^ Department of Medicine, Division of Genetic Medicine Vanderbilt University Medical Center Nashville Tennessee USA; ^17^ Artur Sá Earp Neto University Center ‐ UNIFASE‐FMP, Petrópolis Medical School Rio de Janeiro Brazil; ^18^ Department of Microbiology, Immunology and Physiology School of Medicine, Meharry Medical College Nashville Tennessee USA; ^19^ Department of Biological Sciences Border Biomedical Research Center, the University of Texas at El Paso El Paso Texas USA; ^20^ Department of Pathology Vanderbilt University Medical Center Nashville Tennessee USA; ^21^ Department of Internal Medicine, Section of Gerontology and Geriatric Medicine Sticht Center for Healthy Aging and Alzheimer's Prevention, Wake Forest University School of Medicine Winston‐Salem North Carolina USA; ^22^ Department of Pathology Johns Hopkins University School of Medicine Baltimore Maryland USA; ^23^ Division of Clinical Pharmacology Vanderbilt University Medical Center Nashville Tennessee USA; ^24^ Department of Chemistry Vanderbilt University Nashville Tennessee USA; ^25^ Department of Cell and Developmental Biology Vanderbilt University Nashville Tennessee USA; ^26^ Department of Internal Medicine, Division of Endocrinology and Metabolism Carver College of Medicine, University of Iowa Iowa City Iowa USA; ^27^ Fraternal Order of Eagles Diabetes Research Center Iowa City Iowa USA; ^28^ Department of Molecular and Human Genetics Baylor College of Medicine Houston Texas USA; ^29^ Department of Computer Science Whiting School of Engineering, Johns Hopkins University Baltimore Maryland USA; ^30^ Department of Social and Behavioral Sciences Yale School of Public Health New Haven Connecticut USA; ^31^ Department of Epidemiology Harvard T.H. Chan School of Public Health Boston Massachusetts USA

**Keywords:** 3DEM, kidney, metabolism, MICOS complex, mitochondria

## Abstract

Due to aging, the efficiency of kidney function begins to decrease. Dysfunction in mitochondria and their cristae is a hallmark of aging. Therefore, age‐related decline in kidney function could be attributed to changes in mitochondrial ultrastructure, increased reactive oxygen species, and alterations in metabolism and lipid composition. We sought to understand how mitochondrial ultrastructure is altered over time in tubular kidney cells. A serial block face‐scanning electron microscope and manual segmentation using the Amira software were employed to visualize murine kidney samples during the aging process at 3 months (young) and 2 years (old). We found that 2‐year mitochondria are more fragmented with many uniquely shaped mitochondria observed across aging, concomitant with shifts in ROS, metabolomics, and lipid homeostasis. Furthermore, we demonstrate that the mitochondrial contact site and cristae organizing system (MICOS) complex is impaired in the kidney during aging. Disruption of the MICOS complex resulted in altered mitochondrial metabolic function and increased ROS levels. We found significant, detrimental structural changes in the mitochondria of aged kidney tubules, suggesting a potential mechanism underlying the increased frequency of kidney disease with aging. We hypothesize that disruption of the MICOS complex exacerbates mitochondrial dysfunction, creating a vicious cycle of mitochondrial degradation and oxidative stress, which impacts kidney health.

## Graphical abstract

During kidney aging, loss of the MICOS complex drives cristae disorganization, impairs oxidative capacity, and alters mitochondrial dynamics. MICOS disruption elevates mitochondrial ROS and disrupts calcium homeostasis, linking mitochondrial structural remodeling to metabolic dysfunction in aging kidney tissue.

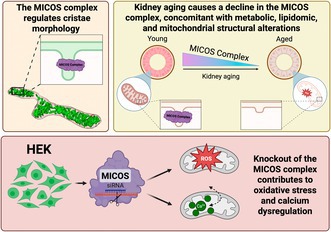

## 1 Introduction

Kidneys are primarily known for their role in excreting waste products from the body. However, their functions extend far beyond this, including hormonal signaling, making the kidney critical for many other functions, such as blood pressure regulation (Ferguson and Waikar 2012). However, renal dysfunction may occur in various states, such as the sudden loss of kidney function, acute kidney injury (AKI), and chronic kidney diseases (CKD) (Ishimoto and Inagi 2016). An estimated 90% of afflicted individuals are not aware that they have an AKI or CKD; thus, the exact prevalence and burden of kidney diseases remain difficult to measure (Chronic Kidney Disease in the United States 2020). Poor treatment outcomes for kidney diseases and their associations with other conditions, including cardiovascular disease, emphasize the importance of developing new, effective treatments for kidney disease (Duann and Lin 2017). Kidneys are among the most mitochondria‐rich tissues in the body (Bhargava and Schnellmann 2017). Therefore, studying the role of mitochondria in kidney function and disease provides an avenue for understanding kidney disease pathophysiology (Bhargava and Schnellmann 2017).

Researchers widely recognize that mitochondria provide numerous critical cellular functions beyond just energy production through oxidative phosphorylation (Glancy 2020). Mitochondria play a crucial role in cell signaling, calcium regulation, apoptosis, and overall cellular homeostasis. Mitochondrial genes also encode the pathways responsible for ATP generation (Picard and McEwen 2014). Mitochondrial dysfunction contributes to kidney diseases (Duchen and Szabadkai 2010; Mao et al. 2024; Parasyri et al. 2022; Forbes and Thorburn 2018), and is also implicated in diseases affecting mitochondria‐rich organs, including muscle diseases (Alway et al. 2017; Hepple 2014), neurological diseases (Flannery and Trushina 2019; Zhang et al. 2016), and obesity‐associated diabetes (Boudina et al. 2007; Friederich et al. 2009). Notably, specific mechanisms of mitochondrial dysfunction that may govern each disease state, as well as ways to rescue mitochondrial function, remain nascent research topics. Therefore, it is essential to investigate the therapeutic implications of mitochondria.

Studies have shown that mitochondrial dysfunction is a hallmark of AKI pathogenesis, making mitochondria a critical target for restoring kidney function to predisease states (Ishimoto and Inagi 2016; Venkatachalam and Weinberg 2012). Other focus areas for kidney research include autosomal dominant polycystic kidney disease, which has been shown to shift mitochondrial function toward anaerobic respiration (Padovano et al. 2018) through various mechanisms, such as calcium signaling, thereby limiting oxidative capacity. The primary roles, frequency, and connections of mitochondria in different tissues may vary significantly. For mitochondrial calcium regulation, the mitochondrial calcium uniporter (MCU), which regulates calcium influx in mitochondria, is more active in the kidney than in other mitochondria‐rich organs, such as the liver and the heart (Fieni et al. 2012). Still, the mechanisms that mediate kidney mitochondrial dynamics remain unclear.

One link between mitochondrial function and kidney diseases is the aging process. Aging is the most significant risk factor for both CKD and AKI (Lesnefsky et al. 2016; Zhang et al. 2021). It is well established that mitochondrial function declines with aging across organ systems (Figueiredo et al. 2008; Lesnefsky et al. 2016; Zhang et al. 2021), with mitochondrial dysfunction now recognized as a hallmark of aging process (Bratic and Larsson 2013). Given the strong association between aging and the incidence of AKI and CKD, age‐associated mitochondrial dysfunction is likely to contribute to the pathogenesis of both AKI and CKD (Figueiredo et al. 2008; Lesnefsky et al. 2016; Zhang et al. 2021). Past research has implicated that proton leak across the inner mitochondrial membrane leads to a loss of mitochondrial bioenergetics, reducing electron transfer chain efficiency in mouse kidneys (Serviddio et al. 2007). Furthermore, clearance of damaged mitochondria through mitophagy responses is also blunted in aging kidney proximal tubules (Yamamoto et al. 2016). However, it is poorly understood by what mechanism mitochondrial function declines with age, as well as the relationship between mitochondrial dysfunction and mitochondrial structural changes in the kidneys. It is possible that, as in other tissues, mitochondrial fusion is decreased with age, leading to damaged mitochondria and the breakdown of cristae. This is supported by previous studies examining aging kidneys via transmission electron microscopy (TEM) (Cui et al. 2012). However, TEM only allows analysis of mitochondria in 2D, which does not provide sufficient detail on mitochondrial subcellular structures (Marshall et al. 2023; Courson et al. 2021). Thus, we performed 3D reconstruction using a serial block face‐scanning electron microscopy (SBF‐SEM), which allows for a broader range of analysis (Marshall et al. 2023; Courson et al. 2021), to determine how mitochondrial networking and structures are altered across aging in mouse kidneys. We studied mice at two ages: 3‐month‐old mice, representing a young adult phenotype, and 2‐year‐old mice, representing a geriatric model (Dutta and Sengupta 2016). We then studied the morphological changes in mitochondria and cristae in both 2D and 3D, as well as mitochondrial reactive oxygen species (ROS) production, in young and aged mice. Mitochondrial ROS synthesis mainly occurs on the electron transport chain located in the inner mitochondrial membrane during the process of oxidative phosphorylation (Li et al. 2017; Tomsa et al. 2019). Damaged or dysfunctional mitochondria are harmful to the cells because they release substances that promote cell death and create ROS, which causes Thymic epithelial cell‐induced apoptosis (Tomsa et al. 2019). The relationships among mitochondrial oxidative stress, ROS production, and mitophagy are closely intertwined, and all are involved in the pathophysiology of AKI (Tomsa et al. 2019).

Past studies have suggested that dysfunction of the MICOS complex, a protein complex that regulates cristae morphology, can lead to oxidative stress (Genin et al. 2016). Given that we have previously found age‐dependent losses in the MICOS complex correlating with mitochondrial structural defects in both skeletal and cardiac muscle tissues, we hypothesized that a similar phenotype may be observed in the kidney (Vue, Neikirk, et al. 2023; Vue et al. 2022). The oxidative stress resulting from MICOS dysfunction during aging could confer oxidative damage and impaired calcium homeostasis, characteristic of age‐related AKI and CKD (Gyurászová et al. 2020; Hill Gallant and Spiegel 2017). We found that deletion of the MICOS genes resulted in mitochondrial structural changes and impairments in mitochondrial calcium regulation in the kidney. Because we demonstrated that aging affects both the structure of the kidneys and mitochondria, as well as MICOS, we investigated the metabolomic and lipidomic changes in aging kidneys to further understand the pathways that may mediate the roles of mitochondria and MICOS in aging kidney vulnerability to injury and disease.

## 2 Methods

### 2.1 Study Cohort

Participants were selected from the *All of Us* Research Program, a US longitudinal cohort established by the National Institutes of Health to advance precision medicine through enrollment of a large and diverse adult population aged ≥ 18 years, with enrollment initiated in 2017. All participants provided written informed consent, authorized access to their electronic health record (EHR), and completed baseline surveys that captured demographic information. Data were accessed through the *All of Us* Researcher Workbench (Controlled Tier) (All of Us Research Program Investigators 2019). This study used the Curated Data Repository version 8 (CDRv8; C2024Q3R4; released February 20, 2026), which includes participants enrolled and consented through October 1, 2023. Among the 633,248 participants aged ≥ 18 years at the time of consent, we identified participants with male or female sex assigned at birth at the time of consent, and with available EHR data and short‐read whole‐genome sequencing (srWGS) data. We excluded participants without EHR or srWGS data and those with assigned sex at birth other than male or female or with missing or declined responses for regression modeling. Gene‐specific rare predicted loss‐of‐function (LoF) carrier cohorts were then defined for CHCHD6 and OPA1 using variants annotated as frameshift, splice‐acceptor, splice‐donor, or stop‐gained with allele frequency ≤ 0.001. The reasons for selecting these candiate genes are given later. Noncarriers met the same age‐at‐consent, EHR, srWGS, and sex‐at‐birth criteria and were excluded if they carried qualifying LoF variants in the target gene. For CHCHD6, the initial eligible case–control cohort comprised 314,519 participants, including 148 LoF carriers and 314,371 noncarriers; after derivation of regression covariates and exclusion of participants with missing covariate data, the final covariate‐complete analytic sample included 180,076 participants, including 86 CHCHD6 LoF carriers and 179,990 noncarriers (Figure S1). For OPA1, the initial eligible case–control cohort likewise comprised 314,519 participants, including 83 LoF carriers and 314,436 noncarriers; the final covariate‐complete analytic sample included 180,076 participants, including 49 OPA1 LoF carriers and 180,027 noncarriers (Figure S2).

### 2.2 Phenome‐Wide Association Study (PheWAS)

We performed gene‐based PheWAS analyses in the *All of Us* Research Program cohort. Carrier status was encoded as the independent variable of interest (case). Covariates were derived using PheTK (v0.1.47), an *All of Us*‐adapted Python framework for phecode‐based phenome‐wide association testing (Tran et al. 2025), and included age at last EHR event, sex at birth, and the first five genetic principal components; participants with missing covariates were excluded to generate a covariate‐complete analytic cohort. International Classification of Diseases, Ninth or Tenth Revision, Clinical Modification (ICD‐9‐CM/ICD‐10‐ CM) diagnosis data were restricted to this cohort and mapped to phecodes using phecode version 1.2 (US ICD mapping). Logistic regression was then performed for each phecode with carrier status as the independent variable of interest and phecode case status as the dependent variable, adjusting for age at last event, sex at birth, and the first five principal components. Analyses were restricted to phecodes with at least two occurrences on distinct dates and at least five phenotype cases. Statistical significance was assessed using Bonferroni correction based on the number of phecodes tested (Denny et al. 2010). In the CHCHD6 analysis, 1722 phecodes were tested. In the OPA1 analysis, 1779 phecodes were tested.

### 2.3 Animal Care and Maintenance

As per the protocols previously described, the care and maintenance of the male C57BL/6J mice conformed to the National Institute of Health's guidelines for the use of laboratory animals (Vue, Neikirk, et al. 2023; Pereira et al. 2017; Vue, Garza‐Lopez, et al. 2023). The University of Iowa's Institutional Animal Care and Use Committee (IACUC) or the University of Washington IACUC approved the housing and feeding of these mice. The mice were anesthetized using a mixture of 5% isoflurane and 95% oxygen. All young and old mice were sourced from the same institution and maintained on an identical standard chow diet composed of ground wheat, ground corn, ground oats, and meat meals.

### 2.4 MICOS Complex GREX Association With Kidney Disease Phenotypes in BioVU

The Vanderbilt Institute for Clinical and Translational Research at Vanderbilt University Medical Center curates BioVU, a biobank of genotype and linked de‐identified electronic health record (EHR) data. The BioVU program has been previously described (Danciu et al. 2014; Roden et al. 2008). Briefly, this opt‐in program collects leftover blood samples during routine patient care visits at clinics across Tennessee and processes them for genotyping or genetic sequencing. BioVU currently houses samples from 344,467 individuals, with ongoing sample collection.

Genotype data for 94,474 individuals were generated on the Illumina Multi‐Ethnic Genotyping Array (MEGAEX) as previously described (Dennis et al. 2021). Quality control procedures included filtering for SNP and individual call rates, sex discrepancies, and excessive heterozygosity. We used principal component analysis to identify individuals of European and African genetic ancestry, using the 1000 Genomes populations as reference (1000 Genomes Project Consortium et al. 2015; Price et al. 2006). After imputation using the Michigan Imputation server and the Haplotype Reference Consortium (HRC) reference panel, genotype data underwent additional quality control procedures, including filtering for imputation quality, minor allele frequency, and Hardy–Weinberg Equilibrium (Das et al. 2016; McCarthy et al. 2016). After removing genetically related individuals, we identified 65,363 individuals of European genetic ancestry and 12,313 individuals of African genetic ancestry for analysis. We calculated the genetically regulated gene expression (GREX) in BioVU individuals using models built from the genotype‐tissue expression (GTEx) version 8 project data, which includes genotype and matched transcriptome data for 838 donors across 49 distinct tissues (*Science* 2020). The best‐performing GREX models, based on the highest r^2^ values from PrediXcan, UTMOST, and JTI approaches, were used to model gene expression for *CHCHD3*, *CHCHD6*, and *OPA1* from BioVU genotype data (Gamazon et al. 2015; Hu et al. 2019; Zhou, Jiang, et al. 2020).

To evaluate correlations between kidney disease phenotypes and *CHCHD3*, *CHCHD6*, and *OPA1* GREX, we extracted kidney disease case status from de‐identified EHR data in BioVU participants. Specifically, we tested phenotypes or phecodes mapped from ICD9/10 (International Classification of Diseases, 9th and 10th editions) billing codes (phenotypes tested: Renal failure NOS, CKD, and kidney replaced by transplant in all individuals and Renal sclerosis and Abnormal results of function study of kidney in individuals of European genetic ancestry only), requiring at least 50 cases for each phenotype analysis. We used previously described mapping of phecodes to ICD9/10 codes, which are available through the PheWAS package in R (version 0.99.5–2 and 3.6.0, respectively) and the online PheWAS catalogue (Carroll et al. 2014; Wu et al. 2019; https://phewascatalog.org/phewas/#home).

We required individuals to have at least two documented instances of the ICD codes on unique dates within the medical record to be considered a case. Controls had no relevant ICD codes in their medical records. Logistic regression analysis was used to examine the association between *CHCHD3*, *CHCHD6*, and *OPA1* GREX (predictor variable) with kidney phenotype case status (outcome variable). Covariates within the regression models included principal components (PC1‐10), sex, current age, median age of the medical record, and genotype batch in the ancestry‐stratified analyses. The most stringent global Bonferroni *p* value adjustment was calculated by correcting the *p* value for all gene‐tissue GREX pairs tested (*n* = 80) and all phenotypes tested (*n* = 5, *p* = 0.05/400). Because the GREX models across tissues are highly correlated, we also generated a less stringent within‐tissue Bonferroni *p* value adjustment, correcting the *p* value for the number of genes tested (*n* = 3) and the number of phenotypes tested (*n* = 5 in European ancestry, *p* = 0.05/15, and *n* = 3 in African ancestry, *p* = 0.05/9). Nominal significance was considered *p* < 0.05.

### 2.5 Human Sample Cohort

All human samples were obtained from Brazilian cohorts in accordance with the CARE (Ethics Appreciation Presentation Certificate) guidelines. Samples from young individuals were collected, and experiments were performed under CAEE number 61743916.9.0000.5281; samples from older individuals were collected under CAEE number 10429819.6.0000.5285.

### 2.6 Immunohistochemistry

Young (4–5 months old) and old (21–23 months old) C57BL/6J mice were maintained on a chow diet. Kidney slices were embedded in OCT, with section processing as previously described (Chu et al. 2020). Nitrotyrosine (MilliporeSigma, #06–284,1:1000), and antimouse IgG‐HRP (Abcam; ab97046) staining were performed to measure oxidative stress. Masson trichrome staining was quantified using representative low‐power images from each kidney section, deconvoluted, and thresholded in ImageJ to calculate the blue area relative to the total tissue area.

### 2.7 SBF‐SEM Processing of Kidney Tissue

SBF‐SEM was performed according to previously defined protocols (Garza‐Lopez et al. 2022; Neikirk et al. 2023; Hinton et al. 2023). Anesthesia was induced in male mice using 5% isoflurane. The extracted kidney tissue was treated with 2% glutaraldehyde in 100 mM phosphate buffer for 30 min, dissected into 1‐mm^3^ cubes, and further fixed in a solution containing 2.5% glutaraldehyde, 1% paraformaldehyde, and 120 mM sodium cacodylate for 1 h. Fixation and subsequent steps were collected onto formvar‐coated slot grids (Pella, Redding, CA), stained and imaged as previously described (Garza‐Lopez et al. 2022; Neikirk et al. 2023; Hinton et al. 2023). This includes tissue washing with 100 mM cacodylate buffer, incubation in a mixture of 3% potassium ferrocyanide and 2% osmium tetroxide, followed by dehydration in an ascending series of acetone concentrations. The tissues were then embedded in Epoxy Taab 812 hard resin. Sectioning and imaging of the sample were performed using a VolumeScope 2 SEM (Thermo Fisher Scientific, Waltham, MA).

### 2.8 Live‐Cell Imaging and Analysis

Live‐cell mitochondrial dynamics were visualized using a Nikon Eclipse Ti2 inverted fluorescence microscope equipped with a Yokogawa CSU‐W1 spinning disk confocal scanner, Hamamatsu Fusion BT camera, SoRa super‐resolution module, environmental chamber, piezo stage controller, and solid‐state lasers (405, 488, 561, and 640 nm), all controlled via NIS‐Elements AR software (version 5.42). Cells cultured in 35‐mm MatTek glass‐bottom dishes were imaged with a 100× Plan Apo Lambda D oil immersion objective (NA 1.45), capturing MitoTracker Orange‐labeled mitochondria through the 561 nm channel at 2.5‐s intervals over 5 min using either standard W1 mode (xy pixel size: 65 nm) or SoRa mode (xy pixel size: 23 nm), with laser power maintained at 5% and 100 ms exposure time per frame to balance phototoxicity concerns with signal quality (SNR ~1.5–2.5). The Perfect Focus System maintained z‐stability throughout all acquisitions. At the same time, high‐resolution z‐stacks (10–20 μm thick) were acquired in SoRa mode with 100 nm step sizes and subsequently enhanced using the Nikon Batch Deconvolution module (version 6.10.02), which implements Blind and Richardson‐Lucy algorithms with 20 iterations and automatic noise estimation.

### 2.9 Assessment of ROS Levels

HEK293 wild‐type cells were cultured in high‐glucose DMEM (4.5 g/L glucose) supplemented with 10% fetal bovine serum and 1% penicillin–streptomycin at 37°C with 5% CO_2_. Cells (0.2 × 10^6^) were plated in 35‐mm dishes and transfected the following day with siRNAs targeting *MIC60* (Thermo Fisher, 136,128) or *CHCHD6* (Thermo Fisher, 34,035) using Lipofectamine RNAiMAX (Invitrogen), according to the manufacturer's instructions. After incubation for 30 h., cells were co‐stained for 30 min at 37°C with two different dyes for ROS detection: MitoBright ROS Deep Red (10 μM, Dojindo Laboratories) for mitochondrial superoxide detection, and DCFDA (10 μM, Invitrogen) for intracellular total ROS detection. Following incubation with staining dyes, cells were washed three times with 1× HBSS, and ROS imaging was performed using a confocal microscope (FV4000, Olympus Life Science).

For mitochondrial H_2_O_2_ imaging, cells were incubated with MitoPY1 (5 μM, Bio‐Techne) for 45 min at 37°C. Cells were then washed with 1× HBSS and imaged using a confocal microscope (FV4000, Olympus Life Science). ImageJ was used for quantifying fluorescence intensities. COS7 cells were cultured in high‐glucose DMEM (4.5 g/L glucose; Gibco) supplemented with 10% fetal bovine serum and 1% penicillin–streptomycin. Cells were treated with a MICOS inhibitor or vehicle control (DMSO) as indicated. Mitochondria were labeled using MitoTracker Orange according to the manufacturer's instructions.

### 2.10 Knockdown of *MIC60* and *CHCHD6* in HEK293 Cells

The *MIC60* and *CHCHD6* siRNAs, along with a scramble siRNA control, were transfected into HEK293 cells using Lipofectamine RNAiMax (Invitrogen) according to the manufacturer's instructions. After a 48‐h incubation, cells were used for calcium measurements.

### 2.11 Measurement of Mitochondrial Ca^2+^ Uptake in HEK293 Cells

Mitochondrial Ca^2+^ uptake was assessed using a multiwavelength excitation dual‐wavelength emission fluorimeter (Delta RAM, Photon Technology Int.) with slight modifications following the protocol outlined in Tomar et al., 2016 (PMID: 27184846). An equal number of cells (2.5 × 10^6^ cells) were uniformly cleansed with Ca^2+^/Mg^2+^‐free DPBS (GIBCO) and subsequently permeabilized in 1 mL of intracellular medium (ICM‐ 120 mM KCl, 10 mM NaCl, 1 mM KH_2_PO_4_, 20 mM HEPES‐Tris, pH 7.2) containing 20 μg/mL digitonin, 1.5 μM thapsigargin to inhibit the SERCA pump and 2.5 mM succinate to energize the mitochondria. The loading of Fura‐FF (1 μM) at the 0 s time point facilitated the measurement of mitochondrial Ca^2+^ uptake. Fluorescence was recorded at 340‐ and 380 nm excitation/510 nm emission, with continuous stirring at 37°C. At specified time points, a bolus of 5 μM Ca^2+^ and the mitochondrial uncoupler FCCP (10 μM) were introduced into the cell suspension.

### 2.12 Assessment of mCa^2^ ^+^ Retention Capacity (CRC)

To assess mCa^2+^ retention capacity (CRC), 2 × 106 cells were resuspended in an intracellular‐like medium containing (120 mM KCl, 10 mM NaCl, 1 mM KH2PO4, 20 mM HEPES‐Tris, pH 7.2), 1.5 μM Thapsigargin (Tg) to inhibit SERCA so that the movement of Ca^2+^ was solely influenced by mitochondrial uptake, 20‐μg/ml Digitonin (Dg), supplemented with 2.5 μM succinate. All solutions were treated with Chelex 100 (Sigma) to remove traces of Ca^2+^. Digitonin‐permeabilized cells were loaded with the ratiometric reporter FuraFF at 1 μM. Fluorescence was recorded using a spectrofluorometer (Delta RAM, Photon Technology International) with excitation at 340 nm and emission at 510 nm. Following baseline recordings, a repetitive series of Ca^2+^ boluses (5 μM) was introduced at the indicated time points. Upon reaching a steady‐state recording, a protonophore, 10 μM FCCP, was added to collapse the Δψm and release matrix‐free Ca^2+^. The number of Ca^2+^ boluses taken up by cells was counted to calculate mitochondrial CRC.

### 2.13 Data Analysis

GraphPad Prism (La Jolla, CA, USA) was used for all statistical analyses. All experiments involving SBF‐SEM and TEM data included at least three independent replicates. Statistics were not handled by the experimenters. The black bars represent the standard error of the mean. For all analyses, one‐way ANOVA was performed with tests against each independent group and significance was assessed using Fisher's protected least significant difference (LSD) test. *, **, ***, **** were set to show significant difference, denoting *p* < 0.05, *p* < 0.01, *p* < 0.001, and *p* < 0.0001, respectively.

## 3 Results

### 3.1 Phenome‐Wide Association Analyses Links CHCHD6 and OPA1 Loss‐Of‐Function to Kidney and Genitourinary Disease Phenotypes

**FIGURE 1:**
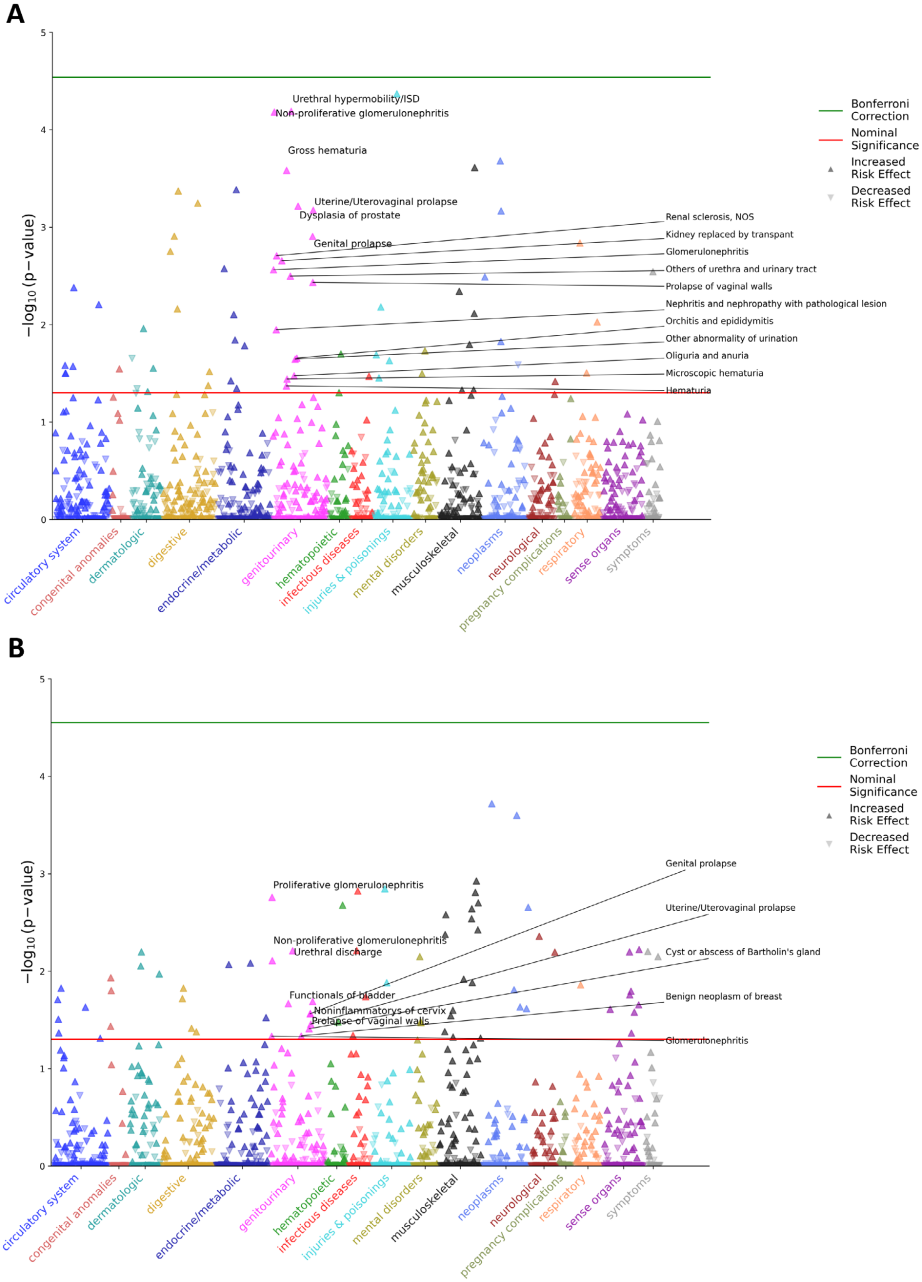
(A) PheWAS Manhattan plot of associations between CHCHD6 loss‐of‐function carrier status and phecode‐defined phenotypes in the *All of Us* cohort. Each point corresponds to a single phecode tested in logistic regression, with CHCHD6 predicted loss‐of‐function carrier status as the independent variable of interest and phecode case status as the outcome. Phenotypes are arranged by clinical category on the *x*‐axis and plotted by −log_10_(*p*‐value) on the *y*‐axis. Upward triangles denote phenotypes with higher odds in carriers, and downward triangles denote phenotypes with lower odds in carriers. The red line indicates nominal significance (*p* < 0.05), and the green line indicates the Bonferroni correction threshold. Nominally significant genitourinary phenotypes are annotated. (B) PheWAS Manhattan plot of associations between OPA1 loss‐of‐function carrier status and phecode‐defined phenotypes in the *All of Us* cohort. Each point corresponds to a single phecode tested in logistic regression, with OPA1 predicted loss‐of‐function carrier status as the independent variable of interest and phecode case status as the outcome. Phenotypes are arranged by clinical category on the *x*‐axis and plotted by −log_10_(*p*‐value) on the *y*‐axis. Upward triangles denote phenotypes with higher odds in carriers, and downward triangles denote phenotypes with lower odds in carriers. The red line indicates nominal significance (*p* < 0.05), and the green line indicates the Bonferroni correction threshold. Nominally significant genitourinary phenotypes are annotated.

We performed a gene‐based phenome‐wide association study (PheWAS) in an *All of Us* cohort to examine the relationship between rare predicted CHCHD6 and OPA1 loss‐of‐function carrier status (the exposure variables) and clinical disease phenotypes. In the CHCHD6 analysis (Figure 1A), a covariate‐complete cohort of 180,076 participants (86 carriers and 179,990 noncarriers) was tested across 1722 phecodes using covariate‐adjusted logistic regression, with two phecodes exceeding the Bonferroni threshold in the full scan. Notably, nominal associations were enriched among renal and genitourinary phenotypes (Table S1), including glomerulonephritis (OR = 8.61; *p* = 2.73 × 10–3), renal sclerosis (NOS) (OR = 9.27; *p* = 1.96 × 10^−3^), kidney replaced by transplant (OR = 6.13; *p* = 2.21 × 10^−3^), nephritis/nephropathy with pathological lesion (OR = 6.16; *p* = 1.13 × 10^−2^), hematuria (OR = 2.02; *p* = 4.26 × 10^–2^), and oliguria/anuria (OR = 8.56; *p* = 3.35 × 10^−2^). In a parallel OPA1 loss‐of‐function carrier PheWAS (Figure 1B), a covariate‐complete cohort of 180,076 participants (49 carriers and 180,027 noncarriers) was tested across 1779 phecodes, and kidney‐related nominal associations were likewise observed (Table S2), including proliferative glomerulonephritis (OR = 24.04, *p* = 1.75 × 10^−3^), nonproliferative glomerulonephritis (OR = 14.95, *p* = 7.77 × 10^−3^), and glomerulonephritis (OR = 7.52, *p* = 4.65 × 10^−2^). The clustering of these nominal associations within kidney‐relevant diagnostic categories supports a potential link between disruption of CHCHD6/MICOS‐ and OPA1‐dependent mitochondrial inner membrane architecture and renal vulnerability.

### 3.2 Genetic Association Analysis Reveals MICOS Complex Components as Potential Mediators of Kidney Disease Susceptibility

**FIGURE 2:**
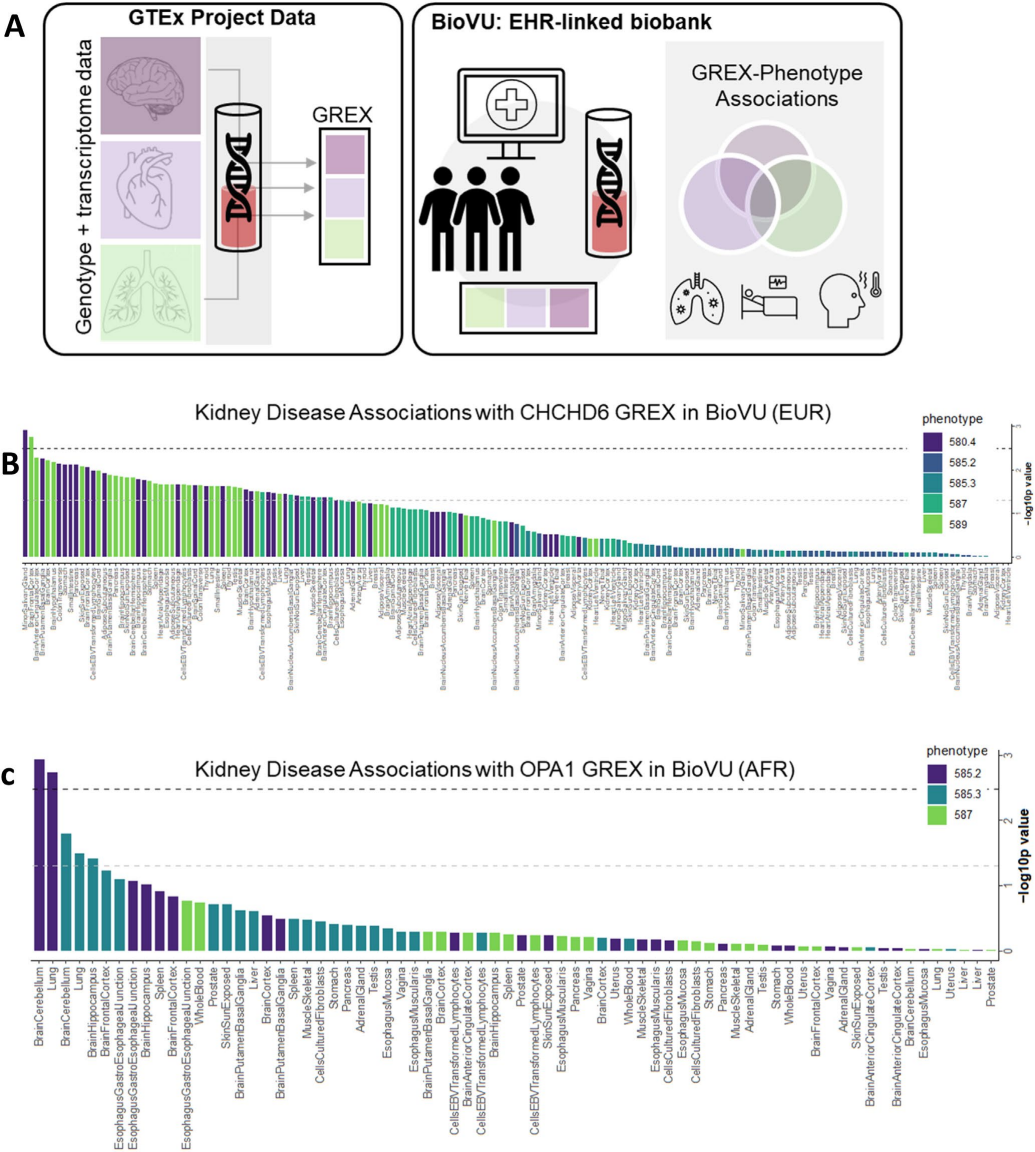
Assessing the relationship between the MICOS complex, genetically modeled gene expression, and kidney diseases in a clinical biobank. (A) Genetically‐regulated gene expression (GREX) for *CHCHD3*, *CHCHD6*, and *OPA1* were calculated in BioVU participants (*n* = 77,676) using models built from the GTEx version 8 data, which contain genotype data matched to transcriptome data from 838 donors across 49 tissues. (left) We identified cases and controls for kidney disease phenotypes from BioVU participants using ICD9/10 codes from Vanderbilt's de‐identified electronic health record database. (right) Genetically modeled expression was tested for association with kidney disease case status using logistic regression models, accounting for genetic ancestry (principal components/PC 1–10), sex, current age, median age of medical record, and genotype batch. (B) Association results for *CHCHD6* GREX and kidney phenotypes in individuals of European ancestry (EUR) are rank‐ordered along the x‐axis by decreasing ‐log10p value. Each bar represents a GREX model with tissues labeled on the x‐axis. The bars are color‐coded according to the kidney phenotype being tested. The black dotted line represents the within‐tissue Bonferroni‐corrected *p*‐value, and the dotted gray line represents the nominal *p*‐value (*p* = 0.05). (C) Association results for OPA1 GREX and kidney phenotypes in individuals of African ancestry (AFR). Legend key: Phecode 580.4: Renal sclerosis, NOS, phecode 585.2: Renal failure NOS, phecode 585.3: Chronic renal failure (CKD), phecode 587: Kidney replaced by transplant, phecode 589: Abnormal results of function study of kidney.

We conducted a comprehensive biobank‐based investigation examining the relationship between genetically determined expression profiles of MICOS complex components and clinical manifestations of kidney disease across a large patient cohort. Leveraging the GTEx v8 database containing matched genotype‐transcriptome data from 838 donors across 49 tissues, researchers calculated genetically‐regulated gene expression (GREX) for key mitochondrial structure regulators (*CHCHD3*, *CHCHD6*, and *OPA1*) in 77,676 BioVU participants (Figure 2A). Kidney disease cases and controls were identified using ICD9/10 diagnostic codes from de‐identified electronic health records, followed by logistic regression analysis to test associations between GREX and kidney disease status while controlling for genetic ancestry (PC1‐10), sex, age variables, and technical factors. The results demonstrate tissue‐specific patterns of association, with *CHCHD6* GREX exhibiting significant relationships with multiple kidney phenotypes in European ancestry individuals (EUR), as visualized in the rank‐ordered tissue‐specific models (Figure 2B). Similarly, for African ancestry individuals (AFR), *OPA1* GREX showed notable associations with renal conditions, including chronic kidney disease, renal failure, kidney transplantation, and abnormal kidney function studies (Figure 2C), suggesting that genetically determined expression levels of mitochondrial structural regulators may influence kidney disease susceptibility and progression.

### 3.3 Human Aging Causes Minimal Changes in Kidney Size

**FIGURE 3:**
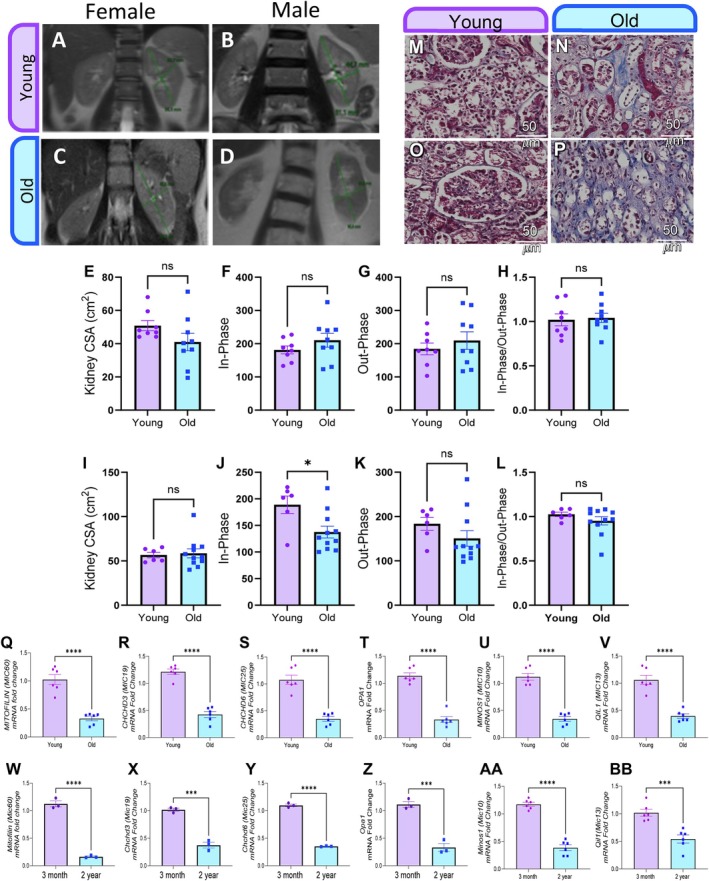
Age‐associated structural, histological, and transcriptional alterations in the human and murine kidney. Cross‐sectional MRI images of the left kidney from (A) females <50 years (14–42 years; *n* = 8), (B) males <50 years (14–48 years; *n* = 6), (C) females > 60 years (65–90 years; *n* = 9), and (D) males > 60 years (60–88 years; *n* = 11). Quantitative MRI analyses are shown separately for females (E–H) and males (I–L). (E, I) Left kidney cross‐sectional area (CSA), calculated as the product of kidney length and width. (F, J) In‐phase signal intensity, reflecting alignment of fat and water magnetic fields. (G, K) Out‐of‐phase signal intensity, reflecting misalignment of fat and water magnetic fields. (H, L) Ratio of in‐phase to out‐of‐phase signal intensities. Representative histological images of human kidney cortex stained with Masson's trichrome (M, O) and hematoxylin & eosin (N, P) from young adults (24–31 years; M, N) and older individuals (60–77 years; O, P). Scale bar, 50 μm. Relative mRNA expression of MICOS complex components in human kidney cortex from young (20–48 years) and old (65–90 years) individuals: (Q) *MIC60*/*MITOFILIN*, (R) *CHCHD3*/*MIC19*, (S) *CHCHD6*/*MIC25*, (T) *OPA1*, (U) *MINOS1*/*MIC10*, and (V) *QIL1*/*MIC13*. Relative mRNA expression of MICOS complex components in murine kidney cortex from 3‐month‐old and 2‐year‐old mice: (W) *Mic60*/*Mitofilin*, (X) *Chchd3*/*Mic19*, (Y) *Chchd6*/*Mic25*, (Z) *Opa1*, (AA) *Minos1*/*Mic10*, and (BB) *Qil1*/*Mic13*. Gene expression was assessed by quantitative PCR, normalized to housekeeping genes, and is presented as mean ± SEM. Statistical significance is indicated as ns, not significant; *p* < 0.05; **p < 0.001; ***p < 0.0001.

Previous studies have utilized magnetic resonance imaging (MRI) of solid renal masses as a proxy for pathologic classification and to define kidney structure (Childs et al. 2014; Outwater et al. 1997). Generally, it has been found that, after the age of 60, kidney volume decreases at approximately 16 cm^3^ per decade (Fang et al. 2020). Thus, we utilized MRI to determine how the kidney is remodeled during the aging process. By enrolling female (Figure 3A,C) and male participants (Figure 3B,D), we created a “young” cohort (*n* = 14) consisting of individuals under 50 years old and an “old” cohort (*n* = 20) of individuals aged 60 or older. For both sexes, the kidney area did not show a significant change (Figure 3E,I). In‐phase, which refers to aligned fat and water molecules, and out‐of‐phase, or opposed‐phase, intensity were similarly minimally changed in both females and males across aging (Figure 3F,G female, Figure 3J,K male). We observed that the old cohort of males had a significantly reduced in‐phase intensity (Figure 3J). From there, we calculated the ratio of in‐phase to out‐of‐phase intensity, which showed no significant difference (Figure 3H,L). When male and female subjects were combined, the kidneys from males and females were not significantly differentiated across the aging process. Interestingly, older females exhibited substantially lower cross‐sectional areas than aged males, suggesting an increased susceptibility to aging with sex‐dependent differences in kidney aging.

Histological examination of human kidney cortex samples revealed distinct structural differences between age groups. Masson's Trichrome staining demonstrated altered extracellular matrix composition and fibrotic changes in older individuals (60–77 years) compared to younger adults (24–31 years) (Figure 3M,N respectively). Complementary H&E staining further elucidated age‐associated alterations in tissue architecture, cellular morphology, and organization within the kidney cortex (Figure 3O,P, young and old, respectively).

### 3.4 Aging Is Associated With Coordinated Downregulation of MICOS Components and Cristae Regulators in Human and Murine Kidneys

To determine whether aging is associated with altered expression of mitochondrial inner membrane organizing factors in vivo, we analyzed transcript levels of MICOS components and key cristae regulators in human kidney samples and in an independent murine aging model. In human kidneys, aging was associated with a robust and coordinated reduction in the expression of multiple MICOS subunits and cristae‐associated genes. Quantitative mRNA analysis revealed a significant decrease in *MIC60 (Mitofilin)* expression in aged samples compared with young controls (Figure 3Q). Similarly, transcripts encoding *CHCHD3 (MIC19)* and *CHCHD6 (MIC25)* were markedly reduced with age (Figure 3R,S). In addition to MICOS core components, expression of the mitochondrial inner membrane fusion regulator *OPA1* was significantly lower in aged human kidneys (Figure 3T). Transcripts corresponding to additional MICOS‐associated subunits, including *MIC10 (MINOS1)* and *MIC13 (QIL1)*, were also significantly downregulated in aged samples (Figure 3U,V), indicating a broad age‐dependent suppression of the MICOS transcriptional program. Although we could not confirm that subjects had kidney disease, these results suggest a slight age‐related decline in kidney structure. Although gross morphological changes may be minimal, we sought to elucidate other tissue changes that occur with aging.

To assess whether this age‐associated MICOS decline is conserved across species, we next examined renal gene expression in a murine aging model comparing young (3‐month‐old) and aged (2‐year‐old) mice. Consistent with the human data, aged mouse kidneys exhibited a significant reduction in *Mitofilin (Mic60)* mRNA levels relative to young animals (Figure 3W). Expression of *Chchd3 (Mic19)* and *Chchd6 (Mic25)* was similarly decreased with age (Figure 3X,Y). In parallel, *Opa1* transcript levels were significantly reduced in aged mice (Figure 3Z), accompanied by decreased expression of *Minos1 (Mic10)* and *Quil1 (Mic13)* (Figure 3AA,BB).

### 3.5 Murine Aging Results in Interstitial Fibrosis and Oxidative Stress

**FIGURE 4:**
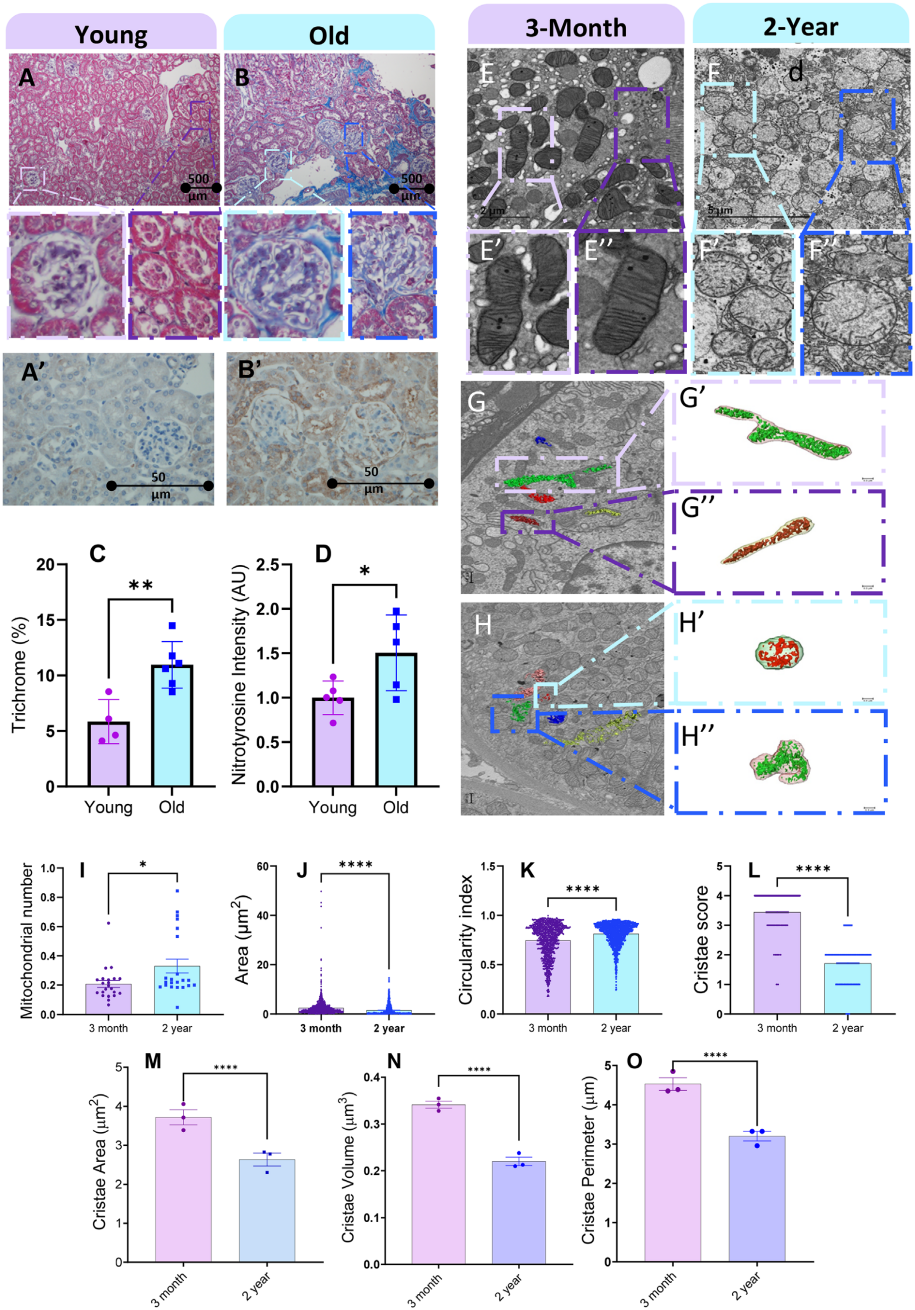
Age‐associated histological and ultrastructural remodeling of renal mitochondria in mice. (A, B) Representative images of Masson's trichrome staining of the kidney cortex from young (4–5 months) and old (21–23 months) mice. Insets show higher‐magnification views of glomerular and tubular regions. (A′, B′) Representative immunohistochemistry for nitrotyrosine (brown) in the kidney cortex from young (4–5 months) and old (21–23 months) mice. (C) Quantification of trichrome‐positive area (%) and (D) nitrotyrosine staining intensity corresponding to panels A′ and B′. (E–E″) Representative transmission electron microscopy (TEM) images of kidney tissue from 3‐month‐old male mice and (F–F″) 2‐year‐old male mice, showing mitochondrial ultrastructure at increasing magnifications. (G, H) Representative serial block‐face scanning electron microscopy (SBF‐SEM) images illustrating three‐dimensional cristae morphology in 3‐month‐old and 2‐year‐old samples, respectively. Boxed regions indicate mitochondria selected for cristae segmentation and magnified in (G′–G″) and (H′–H″). Quantifications in panels I–L were derived from TEM images, whereas cristae morphometric parameters in panels M–O were obtained from three‐dimensional SBF‐SEM reconstructions. (I) Quantification of mitochondrial number (J), mitochondrial area (K), circularity index (L), and cristae score, a semiquantitative measure of observed cristae organization. (M) Cristae area, (N) cristae volume, and (O) cristae perimeter derived from three‐dimensional reconstructions. Mitochondrial measurements were obtained from approximately 1050 mitochondria at 3 months and approximately 1450 mitochondria at 2 years. Cristae score analysis included 1093 mitochondria. Each dot represents an individual mitochondrion, with variable numbers per animal. For TEM‐based analyses, each age group comprised 3 mice. Data are presented as mean ± SEM. Statistical comparisons were performed using Mann–Whitney U tests. Statistical significance is denoted as ns (not significant), *p* < 0.05, *p* < 0.01, and **p ≤ 0.0001.

Previous studies have shown that interstitial fibrosis on kidney biopsy is a prognostic indicator and can represent nephropathy, although its diagnostic effectiveness is mixed (Wang et al. 2023; Menn‐Josephy et al. 2016). We examined young (4–5 months old; Figure 4A) and old (21–23 months old; Figure 4B) C57BL/6J mice using trichrome blue to stain connective tissue. Concurrent with previous studies (Chu et al. 2020), we found that the trichrome area percentage was higher in the old mice (11.0%) than in the young mice (5.8%), indicating that older mice had a greater degree of interstitial fibrosis (Figure 4C). From there, we used immunohistochemistry to examine nitrotyrosine, a biomarker of oxidative stress (Barrera et al. 2016), as shown in the brown areas (Figures 4A′ and 3B′). Studies have shown that increased nitrotyrosine levels correlate with renal dysfunction and inflammatory processes, serving as a biomarker for kidney diseases such as AKI and CKD, as well as overall mortality in these disease states (Lv et al. 2018; Walker et al. 2001; Qian et al. 2013). Immunohistochemistry revealed a significant increase in nitrotyrosine in tubular epithelial cells and podocytes of old mice compared with their young counterparts (Figure 4D). This indicates that oxidative stress occurs with aging, so we sought to understand how mitochondrial structure also changes.

### 3.6 Ultrastructural Analysis Reveals Age‐Associated Enlargement and Rounding of Renal Mitochondria

Previous studies have shown that aging influences mitochondrial dynamics and morphology in the kidney (O'Toole et al. 2010). To assess age‐dependent ultrastructural changes, we analyzed TEM images of renal tubular mitochondria from 3‐month‐ and 2‐year‐old mice (Figure 4E–F″), using samples obtained from three animals per group. Examination of TEM sections revealed clear alterations in mitochondrial morphology with advancing age. Quantitative morphometric analysis demonstrated a significant increase in average mitochondrial area in aged kidneys. Mitochondria from 3‐month‐old mice exhibited a mean area of 1.57 μm^2^ ± 2.09 μm^2^ (SD), whereas mitochondria from 2‐year‐old mice showed a larger mean area of 2.54 μm^2^ ± 3.33 μm^2^ (SD) (Figure 4J). In parallel, mitochondrial shape was altered with aging, as reflected by a significant increase in the circularity index from 0.746 ± 0.174 (SD) in 3‐month‐old mice to 0.813 ± 0.116 (SD) in 2‐year‐old mice (Figure 4K). In contrast, mitochondrial number did not differ significantly between young and aged kidneys (Figure 4I).

Together, these findings indicate that aging is associated with mitochondrial enlargement and increased rounding in renal tubular cells. While increased mitochondrial size could theoretically expand membrane surface area, such morphological changes are also consistent with pathological mitochondrial swelling, a feature commonly linked to mitochondrial stress and dysfunction (Jenkins et al. 2024). Given that mitochondrial ultrastructure, including cristae organization, plays a critical role in regulating bioenergetics (Glancy et al. 2020), these age‐associated morphological alterations prompted further examination of cristae integrity.

### 3.7 Age‐Dependent Loss of Cristae Integrity Revealed by Cristae Scoring and Quantitative Morphometric Analysis

Ultrastructural analysis of TEM images revealed marked age‐associated disruption of cristae organization in renal tubular mitochondria (Figure 4E″,F″, magnified regions). To quantitatively assess these changes, we applied a previously established cristae scoring system (Fang et al. 2020; Hara et al. 2014; Zhou, Long, et al. 2020), an ordinal scale ranging from 0 to 4 that reflects progressive degrees of cristae organization, from little‐to‐no discernible cristae (score 0) to well‐formed, densely packed cristae (score 4). Cristae scoring revealed a significant decline in cristae integrity with aging. Mitochondria from 3‐month‐old mice predominantly exhibited well‐organized cristae, with a mean cristae score of 3.44 ± 0.794 (SD), whereas mitochondria from 2‐year‐old mice showed substantially fewer well‐formed cristae, with a reduced mean score of 1.71 ± 0.693 (SD) (Figure 4L). These results indicate a pronounced loss of cristae structural integrity in aged kidneys.

To complement this qualitative scoring and obtain objective measurements of cristae architecture, we performed morphometric analyses of individual cristae from TEM images. Quantitative assessment revealed a significant reduction in cristae area in aged mitochondria compared with young samples (Figure 4M). In parallel, cristae volume was markedly decreased in 2‐year‐old mice relative to 3‐month‐old controls (Figure 4N), indicating a substantial loss of inner membrane surface area at the level of individual cristae. Consistent with these findings, cristae perimeter was also significantly reduced with aging (Figure 4O), reflecting diminished cristae length and reduced architectural complexity.

Together, cristae scoring, quantitative TEM‐based morphometrics, and complementary SBF‐SEM analyses consistently demonstrate that aging is associated with a profound loss of cristae integrity, size, and complexity in renal mitochondria. Importantly, these defects occur despite an overall increase in mitochondrial size, indicating a decoupling between mitochondrial enlargement and preservation of inner membrane architecture during renal aging.

### 3.8 SBF‐SEM Reveals Aging Results in Reduced Mitochondrial Volume in Kidney Tissue

**FIGURE 5:**
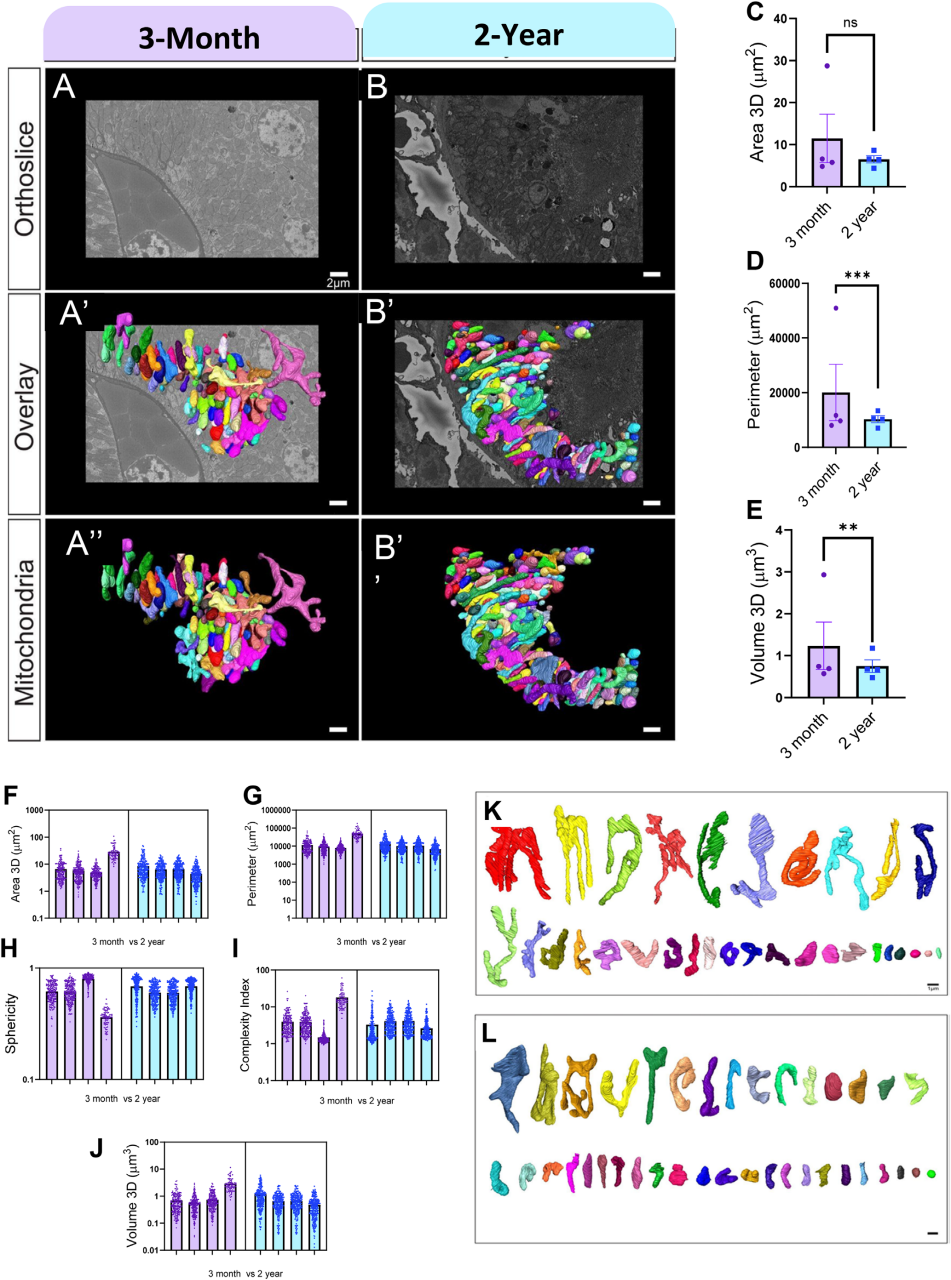
Three‐dimensional mitochondrial morphometry in the aging mouse kidney assessed by SBF‐SEM. (A, B) Representative SBF‐SEM orthogonal (ortho) slices of kidney tissue from 3‐month‐old and 2‐year‐old male mice. (A′, B′) Three‐dimensional reconstructions of mitochondria from 3‐month‐old and 2‐year‐old kidney tissue, respectively, overlaid on corresponding ortho slices. (A″, B″) Isolated three‐dimensional reconstructions of individual mitochondria from 3‐month‐old and 2‐year‐old samples for enhanced visualization. Quantitative analyses of three‐dimensional mitochondrial reconstructions are shown as follows: (C, F) mitochondrial 3D area, (D, G) mitochondrial perimeter, (E, J) mitochondrial volume, (H) sphericity, and (I) complexity index. Each dot represents the average value from a single mouse, with a variable number of mitochondria analyzed per mouse (*n* = 4 mice per age group; 83–251 mitochondria per mouse). In total, 740 mitochondria from 3‐month‐old mice and 962 mitochondria from 2‐year‐old mice were included for statistical analysis. Data are presented as mean ± SEM. Statistical comparisons were performed using the Mann–Whitney test, with significance denoted as ns (not significant), **p ≤ 0.01, and ***p ≤ 0.001. (K, L) Mito‐typing of reconstructed mitochondria from 3‐month‐old (K) and 2‐year‐old (L) mice, arranged by mitochondrial volume to highlight qualitative differences in mitochondrial morphology.

With these observations, we aimed to determine whether mitochondrial networks undergo changes in response to aging. In Figure 4, we show representative orthoslice images of the kidney tissue at each aging point (Figure 5A,B), the overlay of the 3‐D reconstruction on orthoslice (Figure 5A′,B′), and the isolated 3‐D reconstruction (Figure 5A″,B″), with each color representing an independent mitochondrion. We found that the average mitochondrial 3D area, a measure of outer mitochondrial membrane surface area, did not significantly change between 3‐month (mean 8.29 μm^2^ ± 10.1 μm^2^ SD) versus 2‐year (6.46 ± 5.31 μm SD) cohorts, unlike our TEM findings, despite great interindividual heterogeneity (Figure 5C,F). However, the perimeter decreased between the 3‐month (mean 14,328 ± 17,723 μm) and 2‐year (10,241 ± 8273 μm SD) cohorts, and the 2‐year cohort also showed less intra‐individual heterogeneity (Figure 5D,G). This trend toward smaller mitochondria was reflected in the comparison of volume between the 3‐month (0.920 μm^3^ ± 1.06 μm^3^ SD) and 2‐year (0.741μm^3^ ± 0.695 μm^3^ SD) cohorts (Figure 5E,J). Quantitative 3D analysis revealed no significant age‐associated change in mitochondrial sphericity between 3‐month‐ and 2‐year‐old mice (Figure 5H). Mitochondrial complexity index was significantly reduced in 2‐year‐old mice compared with 3‐month‐old controls, indicating a loss of structural intricacy with aging (Figure 5I).

To further elucidate age‐related changes and characterize the mitochondrial types in each age cohort, we employed mito‐otyping, a method similar to karyotyping, to organize mitochondria based on their volumes, thereby enhancing the visualization of overall mitochondrial diversity (Figure 5K,L). Critically, this approach revealed few significant morphological changes, with only branching showing reductions. In combination, the aged kidney mitochondrial morphology resembled that of healthy mitochondria, with a reduced size and lacking a phenotype or fragmentation. Since mitochondria showed a variety of structural changes due to aging, we turned our attention to the MICOS complex as a potential mechanistic regulator of these age‐related changes.

### 3.9 Three‐Dimensional SBF‐SEM Analysis Reveals Age‐Associated Reduction in Mitochondrial Complexity and Mitochondria–ER Contact in Mouse Kidney

**FIGURE 6:**
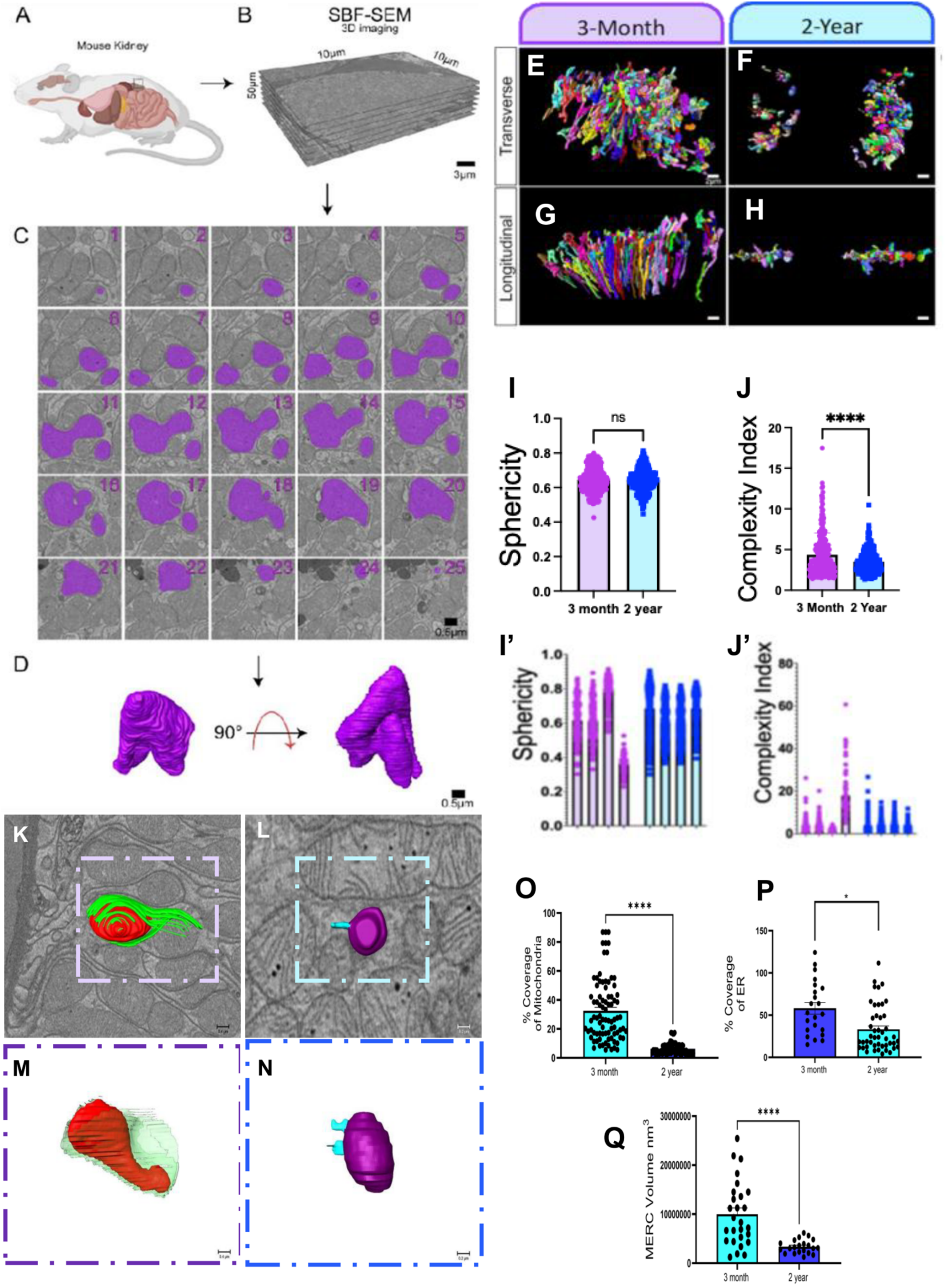
Three‐dimensional analysis of mitochondrial morphology and mitochondria–ER contacts in the aging mouse kidney using SBF‐SEM. (A) Schematic of the experimental workflow illustrating kidney tissue collection from young (3‐month‐old) and aged (2‐year‐old) mice. (B) SBF‐SEM was used to acquire volumetric datasets (50 μm × 10 μm × 10 μm) for three‐dimensional reconstruction. (C) Representative two‐dimensional SBF‐SEM serial sections (25 consecutive slices) from proximal tubule cells, highlighting a segmented mitochondrion (purple). Scale bar, 0.5 μm. (D) Three‐dimensional reconstruction of the mitochondrion shown in (C) with a 90° rotation, illustrating complex membrane topology. Scale bar, 0.5 μm. (E, F) Three‐dimensional reconstructions of mitochondria from 3‐month‐old (E) and 2‐year‐old (F) mouse kidneys are shown in transverse orientation. (G, H) Corresponding longitudinal views of mitochondrial reconstructions from 3‐month‐old (G) and 2‐year‐old (H) samples. Each color represents an individual mitochondrion. Scale bars, 0.5 μm. (I) Quantification of mitochondrial sphericity shows no significant difference between young and aged groups. (J) Quantification of the mitochondrial complexity index demonstrates a significant reduction in aged kidneys compared with young controls. (I′, J′) Distribution of sphericity and complexity index values for individual mitochondria, illustrating greater morphological heterogeneity in young samples and an overall reduction in complexity with aging. (K, L) Representative overlays of three‐dimensional reconstructions of mitochondria and wrapping endoplasmic reticulum (wrappER) on orthogonal SBF‐SEM slices from (K) 3‐month‐old and (L) 2‐year‐old kidneys. (M, N) Side views of three‐dimensional reconstructions of mitochondria and associated wrappER from (M) 3‐month‐old and (N) 2‐year‐old samples. (O) Quantification of the percentage coverage of mitochondria by ER in 3‐month‐old and 2‐year‐old mice. (P) Quantification of ER coverage in young and aged kidneys. (Q) Quantification of mitochondria–ER contact (MERC) volume in 3‐month‐old and 2‐year‐old mice. Data are presented as mean ± SEM. Individual data points represent independent mitochondria reconstructed from SBF‐SEM volumes. Statistical comparisons between age groups were performed using a two‐tailed Mann–Whitney U test. Statistical significance is denoted as ns (not significant), *p* < 0.05, and ***p < 0.0001.

To examine age‐related changes in mitochondrial architecture in kidney proximal tubule cells, we performed high‐resolution three‐dimensional imaging using serial block‐face scanning electron microscopy (SBF‐SEM) on tissue from young (3‐month‐old) and aged (2‐year‐old) mice (Figure 6A–D). Volumetric datasets spanning 50 × 10 × 10 μm were acquired, and individual mitochondria were segmented across 25 consecutive serial sections to generate three‐dimensional reconstructions (Figure 6C,D). Also, due to the substantial time demands of 3D reconstruction and males' higher kidney disease burden from sex differences (Long et al. 2015; Balzer et al. 2022; Agarwal et al. 2013), our study used only a male model.

Three‐dimensional reconstructions revealed clear differences in mitochondrial organization between young and aged kidneys. In 3‐month‐old mice, mitochondria exhibited elongated and highly branched morphologies in both transverse and longitudinal orientations (Figure 6E,G). In contrast, mitochondria from 2‐year‐old mice appeared more fragmented and less elaborately branched (Figure 6F,H). Quantitative analysis of mitochondrial sphericity demonstrated no significant difference between young and aged groups (Figure 6I,I′), indicating that overall mitochondrial roundness is preserved with aging. In contrast, quantification of the mitochondrial complexity index revealed a significant reduction in aged kidneys compared with young controls (Figure 6J,J′), consistent with a loss of three‐dimensional structural complexity. Distribution analysis further showed greater heterogeneity and higher complexity values in mitochondria from young kidneys, whereas mitochondria from aged kidneys clustered toward lower complexity values.

To assess age‐associated changes in mitochondria–endoplasmic reticulum (ER) interactions, we examined three‐dimensional overlays of mitochondria and wrapping ER (wrappER) on orthogonal SBF‐SEM slices. In young kidneys, mitochondria frequently displayed extensive ER coverage consistent with a wrappER phenotype (Figure 6K,M). In contrast, mitochondria from aged kidneys showed a visibly reduced association with the ER (Figure 6L,N). Quantitative analysis confirmed a significant reduction in the percentage of mitochondrial surface covered by ER in aged mice compared with young controls (Figure 6O). Similarly, overall ER coverage within the analyzed volumes was reduced in aged kidneys (Figure 6P). Consistent with these findings, quantification of mitochondria‐ER contact (MERC) volume revealed a significant decrease in aged mice relative to young animals (Figure 6Q). The wrappER‐associated morphology observed in young kidneys resembles ER‐mitochondria interaction phenotypes previously described in other tissues (Jankauskas et al. 2018).

### 3.10 Global Metabolic and Lipid Profiling Highlights Dynamic Changes in the Aged Kidney

**FIGURE 7:**
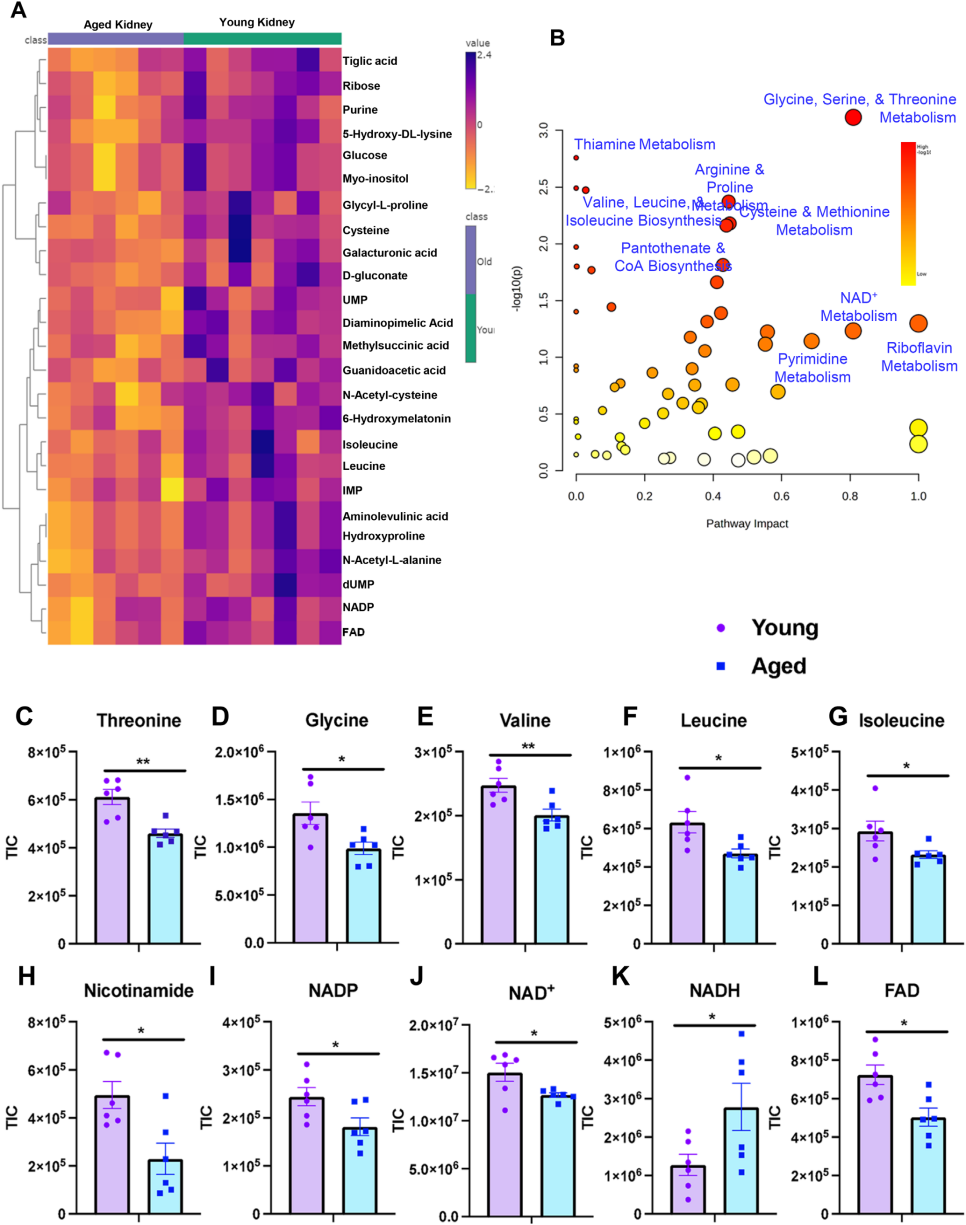
Targeted LC–MS profiling reveals age‐associated metabolic remodeling in kidney tissues. (A–L) Targeted metabolite pool sizes highlighting pathways altered with age, including purine metabolism, amino acid metabolism/biosynthesis, and redox/NAD^+^ metabolism. (A–B) Amino acid metabolite pools show significant age‐related changes. (B) Purine metabolism intermediates, including IMP, UMP, ribose‐phosphate, and purine, are significantly reduced in aged kidneys. (C–G) Amino acid metabolites, including methionine, valine, threonine, leucine, isoleucine, and glycine, are decreased with age. (H–L) Redox and NAD^+^ metabolism intermediates, including nicotinamide, NADP, NAD^+^, NADH, and FAD, demonstrate age‐associated imbalance characterized by reduced NAD^+^, NADP, and FAD levels and increased NADH. Metabolite abundances are presented as total ion counts (TIC), as indicated on the y‐axes. Young, *n* = 6; aged, *n* = 6. Data are shown as mean ± SEM. Statistical significance was determined using Student's t‐test; *p < 0.05, **p < 0.01.

Following our observations of age‐associated mitochondrial structural dysregulation, we sought to define how these morphological changes impact metabolic homeostasis in the kidney. To this end, we performed comprehensive metabolic and lipid profiling of young and aged kidney tissues. Metabolic profiling revealed broad disruptions in amino acid metabolism, nucleotide biosynthesis, and redox cofactors in aged kidneys (Figure 7A–L). Amino acids are central to mitochondrial function, supporting energy production, gluconeogenesis, nitrogen metabolism, antioxidant defense, and specialized mitochondrial pathways (Li and Hoppe 2023). In aged kidneys, we detected significant reductions in methionine, valine, threonine, leucine, isoleucine, and glycine. Notably, glycine, serine, and threonine metabolism—key components of one‐carbon metabolism—were markedly perturbed (Figure 7C). These pathways are tightly linked to mitochondrial function and purine biosynthesis, and disruptions may reflect impaired flux through the mitochondrial glycine cleavage system (GCS), influencing synthesis of purines, pyrimidines, and other small molecules (Figure 7A–B). Consistent with this, we observed significant declines in purine intermediates, including inosine monophosphate (IMP), uridine monophosphate (UMP), ribose‐phosphate, and related purine metabolites, suggesting compromised nucleotide biosynthetic capacity in aged kidneys (Figure 7D–G). Amino acid metabolites, including methionine, valine, threonine, leucine, isoleucine, and glycine, decrease with age (Figure 7C–G). Redox and NAD^+^ metabolism intermediates, including nicotinamide, NADP, NAD^+^, NADH, and FAD, show an age associated imbalance, marked by reduced NAD^+^, NADP, and FAD levels and increased NADH (Figure 7H–L). These findings indicate that despite upstream perturbations in nucleotide and amino acid metabolism, steady‐state ATP levels remain buffered.

To further interrogate mitochondrial energy metabolism, we examined redox cofactors. Aged kidneys exhibited significant declines in NAD^+^, NADP, nicotinamide (NAM), and FAD levels, alongside an increase in NADH (Figure 7B, H–L). This shift suggests an imbalance in redox state consistent with reductive stress. Importantly, although total adenylate pools were unchanged, the altered NAD^+^/NADH ratio and decreased FAD indicate impaired oxidative capacity and electron carrier availability (Figure 7B, H–L). Together, these findings suggest two nonmutually exclusive interpretations: (1) aged kidneys engage compensatory mechanisms that preserve ATP levels despite upstream metabolic remodeling, or (2) metabolic rewiring follows a temporal hierarchy, wherein redox imbalance and nucleotide biosynthetic deficits precede overt ATP depletion (Figure 7A–B). These metabolic signatures align with the mitochondrial morphological and transcriptional alterations observed in our imaging and gene expression analyses, supporting a model in which structural mitochondrial remodeling drives redox and biosynthetic perturbations prior to energetic collapse. Importantly, these measurements were performed in bulk kidney tissue. Thus, compartment‐specific alterations—particularly within mitochondrial ATP pools—may be masked. We cannot definitively conclude whether mitochondrial‐localized ATP production is preserved, as cytosolic buffering mechanisms may obscure organelle‐specific deficits.

We profiled the kidney lipidome in young and old mice to assess age‐associated differences in lipid composition. Principal component analysis showed modest structure by age, with a partial shift in the distribution of samples along the first two components but substantial overlap between groups. Consistent with this, differential abundance analysis at the level of individual lipid species did not reveal a clear statistical signal after correction for multiple testing, with adjusted *p* values remaining above conventional significance thresholds across the dataset (Table S3, Figure Volcano), indicating that age‐related differences are not driven by large changes in a small number of individual molecules.

At the level of lipid classes and structural groupings, lipid set enrichment analysis did identify some coordinated differences between the groups (Enrichment Figures, Table S3). Cardiolipins (CL) were positively enriched in young kidneys (ES = 0.465, *p*_adj_ = 3.22 × 10^−4^), along with related mitochondrial lipid sets including dilysocardiolipins (DLCL; ES = 0.737, *p*_adj_ = 0.0071) and lysophosphatidylinositols (LPI; ES = 0.909, *p*_adj_ = 0.0084). Triglycerides (TG; ES = −0.360, *p*_adj_ = 1.91 × 10^−5^), and N‐acyl ethanolamides (NAE; ES = −0.801, *p*_adj_ = 2.05 × 10^−5^) were negatively enriched, indicating relatively higher representation in older kidneys (Table S4). With respect to structural features, several chain length–defined bins were significantly enriched in young kidneys, including total_cs_8 (ES = 0.531, *p*_adj_ = 3.22 × 10^−4^), total_cl_60 (ES = 0.708, *p*_adj_ = 0.0046), and total_cl_62 (ES = 0.757, *p*_adj_ = 0.0046), while shorter‐chain groupings showed relative depletion.

**FIGURE 8:**
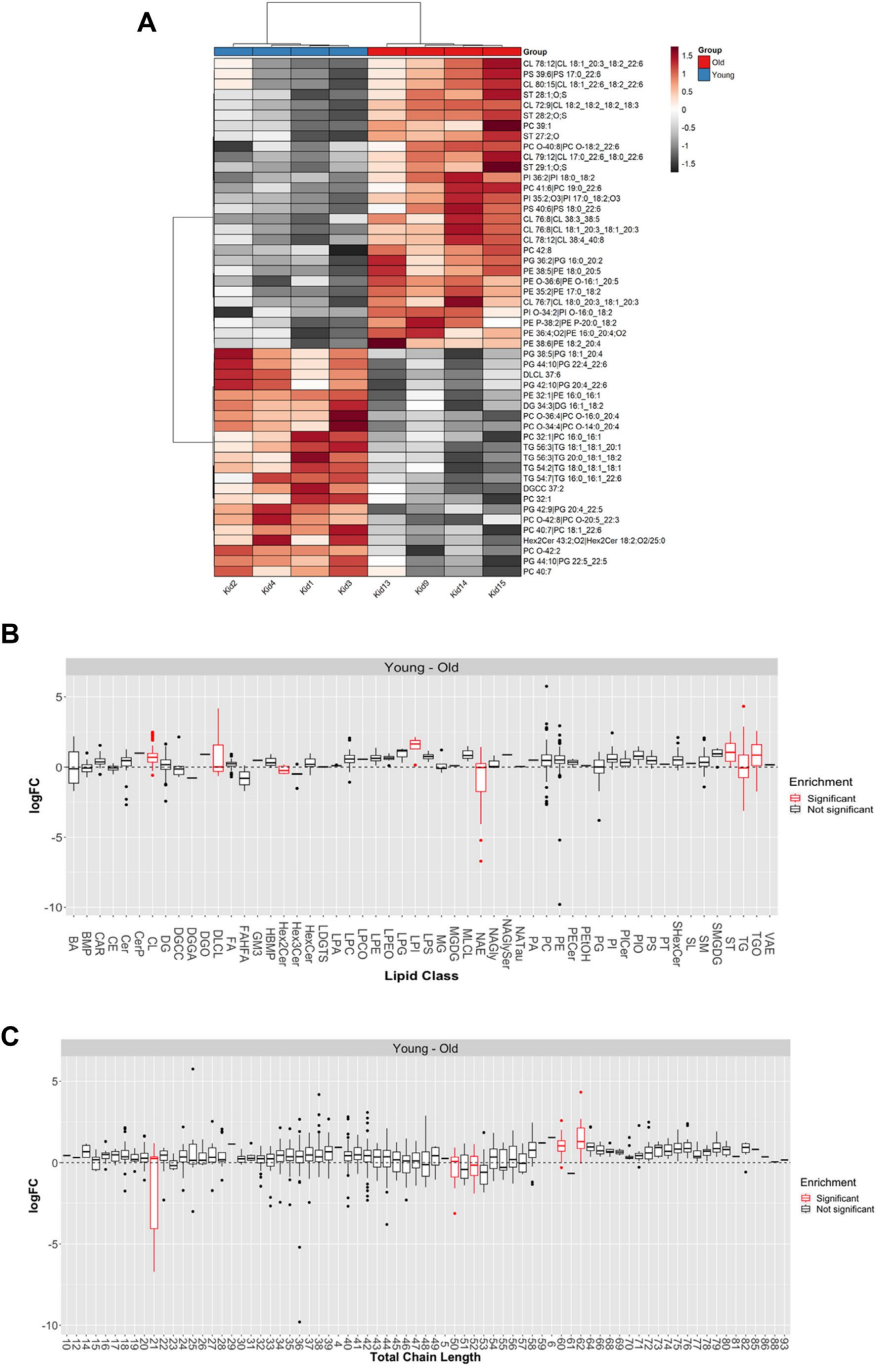
Aging drives coordinated remodeling of lipid classes and acyl chain composition in the mouse kidney (A) Heatmap depicting the relative abundance of lipid species in kidney tissue from young and aged mice. Lipid intensities were normalized across samples and log_2_‐transformed prior to hierarchical clustering. Columns represent individual biological replicates (young, *n* = 6; aged, *n* = 6), and rows represent individual lipid species grouped by class. The color scale indicates relative abundance (log_2_ fold change), with red denoting higher abundance and gray to black denoting lower abundance relative to the dataset mean, as shown in the accompanying scale bar. The top annotation indicates the age group. (B) Lipid class enrichment analysis showing the distribution of log fold change (logFC; young minus old) for individual lipid species within each lipid class. Boxes represent the interquartile range with the median indicated; whiskers denote 1.5× interquartile range; individual points represent lipid species. Lipid classes highlighted in red indicate statistically significant enrichment after multiple‐testing correction. (C) Lipid chain‐length enrichment analysis showing logFC (young minus old) for lipid species grouped by total acyl chain length. Boxes and whiskers are defined as in (B). Chain lengths highlighted in red indicate statistically significant enrichment following multiple‐testing correction. Significantly altered lipid classes and chain lengths were defined as those with false discovery rate (FDR)–adjusted *p* < 0.05 and absolute logFC > 1. Young and aged groups each consisted of *n* = 6 biological replicates. Data are presented as mean ± SEM where applicable. For metabolite‐level comparisons, statistical significance was assessed using an unpaired two‐tailed Student's *t*‐test with FDR correction for multiple comparisons. Statistical significance is denoted as *p* ≤ 0.05 (*) and *p* ≤ 0.01 (**).

Lipidomic profiling of young and aged kidney tissues further revealed age‐dependent remodeling of lipid classes and acyl chain composition (Figure 8A–C). In aged kidneys, significant alterations were observed in triglyceride oligomers (TGO), triglycerides (TG), sterols (ST), N‐acylethanolamines (NAE), lysophosphatidylinositol (LPI), dihexosylceramides (Hex2Cer), dilysocardiolipin (DLCL), and cardiolipin (CL) compared to young controls (Figure 8B). Notably, cardiolipin and dilysocardiolipin are critical structural phospholipids of the inner mitochondrial membrane that stabilize respiratory chain supercomplexes and maintain cristae architecture. Age‐associated alterations in cardiolipin abundance and acyl chain composition likely impair electron transport chain (ETC) organization and efficiency. Because FAD and NAD^+^ function as essential electron carriers feeding into the ETC, structural membrane perturbations may reduce effective electron flux, contributing to the observed decline in NAD^+^ and FAD pools and accumulation of NADH. Thus, mitochondrial membrane remodeling in aged kidneys provides a structural framework that may underlie the redox imbalance consistent with reductive stress. In addition, significant differences in lipid chain length with age (Figure 8C) suggest altered membrane fluidity and curvature, which can further disrupt mitochondrial bioenergetics, calcium handling, and reactive oxygen species signaling. Together, these lipidomic changes support a model in which age‐dependent membrane remodeling compromises mitochondrial respiratory efficiency, reinforcing the redox and metabolic rewiring observed in our metabolic profiling analysis.

### 3.11 Loss of OPA1 or MICOS Components Disrupts Mitochondrial Ultrastructure and Morphology

Oxidative stress is a defining feature of kidney aging and CKD, with mitochondria representing a major cellular source of reactive oxygen species through the electron transport chain (Vue, Neikirk, et al. 2023; Tian et al. 2018; Paradies et al. 2019). Cristae architecture is a key determinant of mitochondrial respiratory efficiency and redox balance and is maintained by the mitochondrial contact site and cristae organizing system (MICOS) (Ilter et al. 2023; Bester et al. 2011; McReynolds et al. 2021, 2020). We previously observed an age‐associated decline in transcripts encoding core MICOS components—*Mic60* (Mitofilin), *Chchd3* (MIC19), and *Chchd6* (MIC25)—and the mitochondrial fusion regulator *Opa1* in kidney tissue (Yan 2021; Lauritzen et al. 2015; Pereira et al. 2017; Mapuskar et al. 2023). These observations prompted us to test whether loss of MICOS integrity, independently or in parallel with OPA1, is sufficient to disrupt mitochondrial ultrastructure and promote oxidative stress (Console et al. 2020).

**FIGURE 9:**
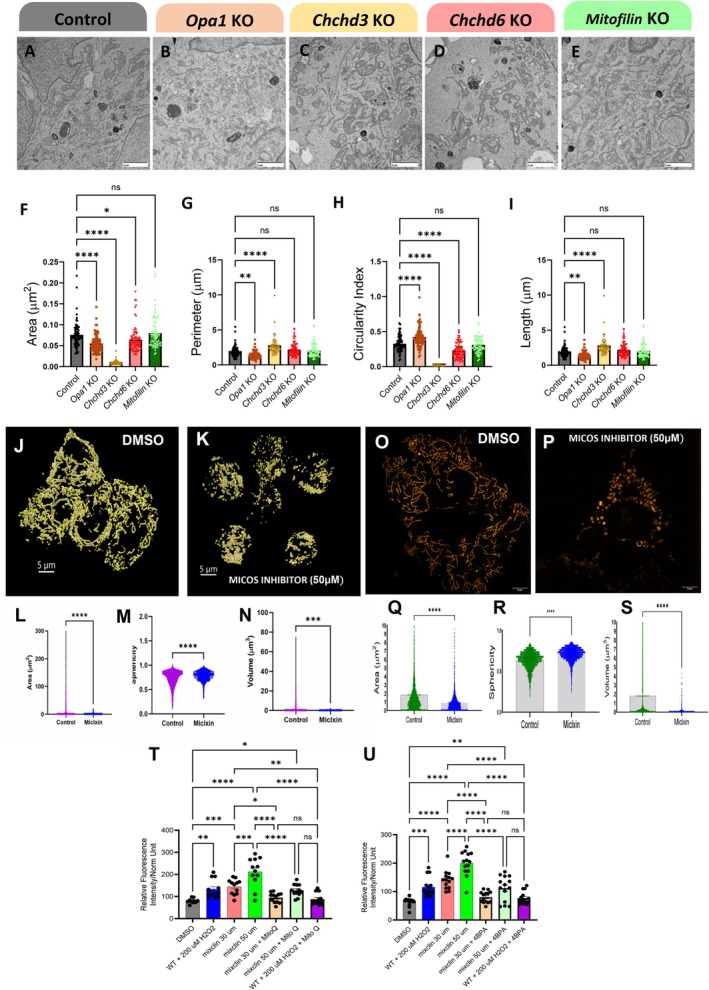
Genetic and pharmacological disruption of MICOS–OPA1 signaling remodels mitochondrial architecture across models and alters redox homeostasis in vitro. (A–E) Representative transmission electron microscopy (TEM) images of mitochondria from kidney tissue of control, *Opa1* knockout (KO), *Chchd3* KO, *Chchd6* KO, and *Mitofilin* KO samples. Control mitochondria display intact cristae organization, whereas loss of OPA1 or MICOS components results in disrupted cristae architecture and altered mitochondrial morphology. Scale bars as indicated. (F–I) Quantitative morphometric analysis of mitochondrial ultrastructure corresponding to panels A–E, including mitochondrial area (F), perimeter (G), circularity index (H), and length (I). Each data point represents an individual mitochondrion; bars indicate mean ± SEM. Statistical significance is denoted as ns (not significant), *p* ≤ 0.05, *p* ≤ 0.01, and **p ≤ 0.0001. (J–S) Pharmacological perturbation of MICOS in cultured cells recapitulates mitochondrial fragmentation phenotypes observed in kidney tissue. Representative three‐dimensional mitochondrial reconstructions from HEK293 cells (J, K) and COS7 cells (O, P) treated with vehicle control (DMSO) or the MICOS inhibitor Miclixin (50 μM). Vehicle‐treated cells exhibit interconnected mitochondrial networks, whereas MICOS inhibition induces mitochondrial fragmentation and loss of network connectivity. Scale bars, 5 μm. Quantitative analyses of mitochondrial area, sphericity, and volume are shown for HEK293 cells (L–N) and COS7 cells (Q–S). Individual data points represent mitochondria; bars indicate mean ± SEM. For all panels, statistical significance is denoted as ns (not significant), *p* ≤ 0.05, *p* ≤ 0.01, *p ≤ 0.001, and **p ≤ 0.0001. Mitochondrial superoxide production was quantified using the MitoSOX Deep Red fluorescent probe and measured by a plate reader. Fluorescence intensity was normalized to control conditions. (T) Relative MitoSOX fluorescence following treatment with vehicle (DMSO), hydrogen peroxide (H_2_O_2_; 200 μM), and increasing doses of the indicated compound (30 μM and 50 μM), either alone or in combination with H_2_O_2_. (U) Independent experimental replicate showing consistent modulation of mitochondrial ROS across treatment conditions. H_2_O_2_ exposure significantly increased mitochondrial superoxide levels, whereas compound treatment reduced MitoSOX fluorescence in a dose‐dependent manner. Co‐treatment attenuated H_2_O_2_‐induced mitochondrial ROS, with no significant differences observed between selected conditions as indicated (ns). Data are presented as mean ± SEM, with each dot representing an independent biological replicate. Statistical significance was assessed by one‐way ANOVA with multiple‐comparison correction; *p* < 0.05, *p* < 0.01, *p ≤ 0.001, and **p ≤ 0.0001; ns, not significant.

Since the loss of *Opa1* triggers morphological changes, we used it as a positive control for morphological changes (Barrera et al. 2016; Gilkerson et al. 2021). We performed siRNA deletions of *Chchd3*, *Mitofilin*, *Chchd6*, and *Opa1* in immortalized human embryonic kidney cells (HEK293 cells). From there, we performed TEM in each condition and compared them with a control (Figure 9A–E). As expected, *Opa1* deletion resulted in significant decreases in mitochondrial area, perimeter, and length, accompanied by an inverse increase in circularity index, which was expected due to impaired fusion dynamics (Figure 9F–I). *Chchd3* deletion exhibits a more drastic phenotype, characterized by a reduced mitochondrial area, whereas *Chchd6* deletion shows a smaller decrease compared with the control. *Mitofilin* deletion demonstrated no significant differences (Figure 9F). Interestingly, *Chchd3* deletion in HEK293 cells results in increased perimeter and length, despite decreased area and circularity index (Figure 9F–I). *Chchd3* KO cells had nearly completely elongated mitochondria, unlike those in *Opa1* deletion. Taken together, this suggests that, while the MICOS complex KO phenotype is distinct from OPA1 loss, it can, beyond its canonical roles in cristae integrity, also modulate mitochondrial structure (Friedman et al. 2015). Since cristae and mitochondrial dysfunction were hallmarks of aging kidneys, we sought to understand the further functional implications of the age‐dependent loss of the MICOS complex.

### 3.12 Pharmacologic MICOS Inhibition Induces Conserved Mitochondrial Fragmentation and Alters Mitochondrial Redox Status

To determine whether acute pharmacologic disruption of MICOS recapitulates mitochondrial structural alterations observed upon genetic perturbation, we treated HEK293 cells with the MICOS inhibitor Miclixin (50 μM) and performed three‐dimensional mitochondrial reconstruction. Vehicle‐treated cells displayed interconnected mitochondrial networks with extensive branching (Figure 9J), whereas Miclixin‐treated cells exhibited fragmented mitochondrial structures with reduced network connectivity (Figure 9K). Quantitative morphometric analysis revealed a significant reduction in mitochondrial area following MICOS inhibition compared with DMSO‐treated controls (Figure 9L). In parallel, mitochondrial sphericity was significantly increased in Miclixin‐treated cells (Figure 9M), indicating a shift toward more rounded mitochondrial morphology. Mitochondrial volume was also significantly reduced upon MICOS inhibition (Figure 9N), consistent with a loss of mitochondrial mass and network integrity.

To assess whether MICOS‐dependent mitochondrial remodeling is conserved across cell types, we performed analogous experiments in COS7 cells treated with Miclixin (50 μM). Three‐dimensional reconstructions showed that vehicle‐treated COS7 cells maintained elongated, reticulated mitochondrial networks (Figure 9O), whereas MICOS inhibition led to pronounced mitochondrial fragmentation and reduced connectivity (Figure 9P). Quantitative analysis showed a significant decrease in mitochondrial area in Miclixin‐treated COS7 cells compared with controls (Figure 9Q). Mitochondrial sphericity was significantly increased following MICOS inhibition (Figure 9R), indicating altered mitochondrial shape. In addition, mitochondrial volume was significantly reduced in Miclixin‐treated cells compared with vehicle‐treated controls (Figure 9S). These findings mirror the structural alterations observed in HEK293 cells, indicating a conserved requirement for MICOS integrity in maintaining mitochondrial morphology.

To examine the functional consequences of MICOS perturbation on mitochondrial redox status, mitochondrial superoxide levels were quantified using the MitoSOX Deep Red probe. Exposure to hydrogen peroxide (H_2_O_2_; 200 μM) significantly increased mitochondrial superoxide production relative to vehicle‐treated controls (Figure 9T). Treatment with the indicated compound at 30 and 50 μM concentrations reduced MitoSOX fluorescence in a dose‐dependent manner (Figure 9T). Co‐treatment with H_2_O_2_ and the compound attenuated H_2_O_2_‐induced mitochondrial superoxide levels, with no significant differences observed between selected treatment conditions as indicated (ns). An independent experimental replicate demonstrated consistent modulation of mitochondrial ROS across treatment groups (Figure 9U), confirming the reproducibility of the observed effects.

### 3.13 Pharmacologic Disruption of Mitochondrial Inner Membrane Organization co‐Ordinately Remodels Organelle Morphology and Interorganelle Interactions

**FIGURE 10:**
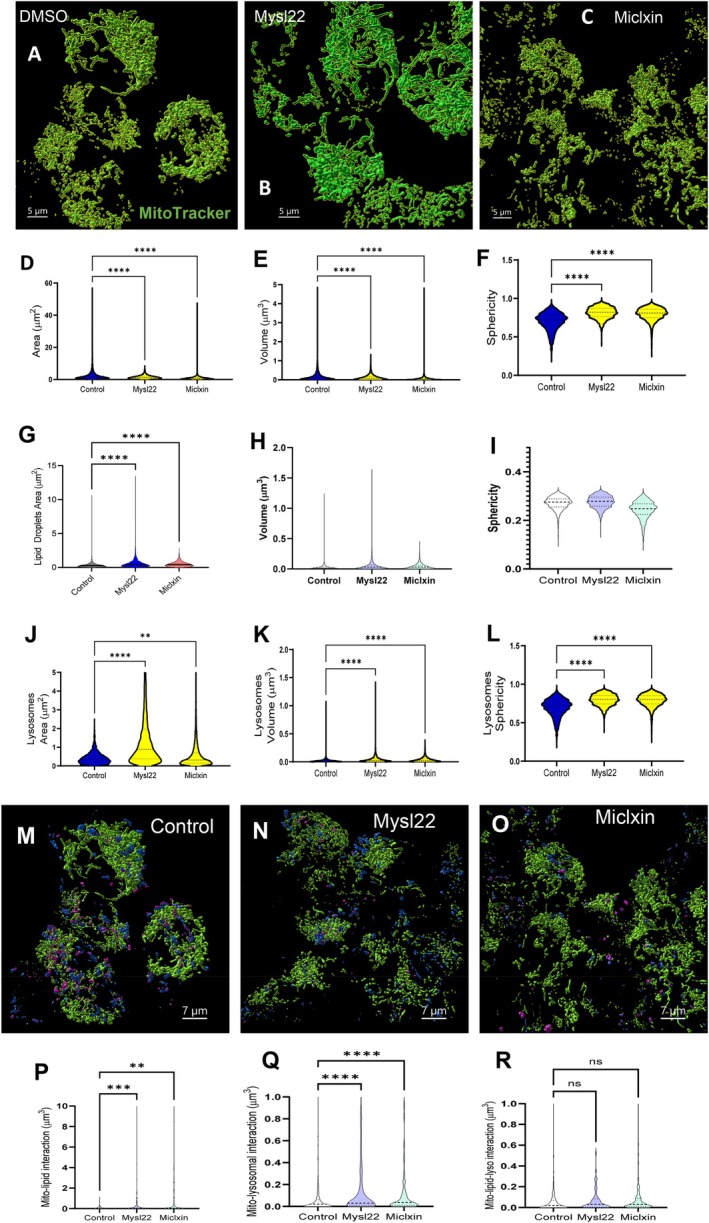
Pharmacological perturbation of mitochondrial inner membrane organization remodels mitochondrial, lipid droplet, and lysosomal morphology and inter‐organelle interactions in HEK293 cells. (A–C) Representative 3D confocal reconstructions of mitochondria labeled with MitoTracker (green) in cells treated with DMSO (control) (A), the OPA1 inhibitor Mysl22 (B), or the MICOS complex inhibitor Miclxin (C). Scale bar, 5 μm. (D–F) Violin plot quantification of mitochondrial area (μm^2^) (D), volume (μm^3^) (E), and sphericity (F), revealing significant remodeling of mitochondrial size and shape following pharmacological inhibition of OPA1 or MICOS compared to control. (G–I) Lipid droplet morphometric analysis showing lipid droplet area (μm^2^) (G), volume (μm^3^) (H), and sphericity (I), indicating altered lipid storage and droplet morphology upon disruption of mitochondrial inner membrane organization. (J–L) Lysosomal morphometrics, including lysosome area (μm^2^) (J), volume (μm^3^) (K), and sphericity (L), demonstrate coordinated changes in lysosomal structure following Mysl22‐ or Miclxin‐mediated perturbation. (M–O) Representative 3D reconstructions of mitochondria (green), lipid droplets (magenta), and lysosomes (blue) in control cells (M), following OPA1 inhibition with Mysl22 (N), or MICOS disruption with Miclxin (O). Scale bar, 7 μm. (P) Quantification of mitochondria–lipid droplet (mito–LD) interactions, showing a significant increase in organelle contacts following Mysl22 treatment relative to control. (Q) Quantification of mitochondria–lysosome (mito–lys) interactions, demonstrating enhanced contacts upon Mysl22 treatment, whereas Miclxin exhibits distinct or non‐significant effects depending on the interaction type. (R) Quantification of three‐way mitochondria–lipid droplet–lysosome (mito–LD–lys) interactions, defined as spatially coincident contacts in which a mitochondrion simultaneously engages both a lipid droplet and a lysosome within the same local volume. All violin plots represent pooled single‐organelle measurements across cells from independent experiments. Statistical significance was assessed using one‐way ANOVA with appropriate multiple‐comparison correction; ns, not significant; **p < 0.01; ***p < 0.001; ****p < 0.0001.

To examine how perturbation of mitochondrial inner membrane organization influences cellular organelle architecture, we analyzed mitochondrial morphology and its relationship with lipid droplets and lysosomes following treatment with Myls22 or Miclxin. Three‐dimensional confocal reconstructions revealed pronounced alterations in mitochondrial organization compared with DMSO‐treated controls (Figure 10A–C). Quantitative morphometric analysis demonstrated significant changes in mitochondrial area, volume, and sphericity upon treatment with either compound, indicating extensive remodeling of mitochondrial size and shape (Figure 10D–F).

Given the central role of mitochondria in lipid metabolism, we next assessed whether mitochondrial inner membrane perturbation was associated with changes in lipid droplet morphology. Both Myls22 and Miclxin treatments resulted in significant alterations in lipid droplet area, volume, and sphericity compared with control cells (Figure 10G–I), consistent with a reorganization of cellular lipid storage properties in response to mitochondrial structural disruption. We further examined lysosomal morphology to determine whether mitochondrial inner membrane perturbation was accompanied by changes in the degradative compartment. Quantitative analysis revealed significant differences in lysosome area, volume, and sphericity following Myls22 and Miclxin treatment relative to controls (Figure 10J–L), suggesting coordinated remodeling of lysosomal structure alongside mitochondrial and lipid droplet alterations.

To investigate whether these morphological changes were accompanied by altered inter‐organelle interactions, we performed three‐dimensional interaction analyses between mitochondria, lipid droplets, and lysosomes. Representative reconstructions under control conditions and following OPA1 or MICOS perturbation illustrate distinct patterns of organelle proximity and organization (Figure 10M–O). Quantitative analysis revealed a significant increase in mitochondria–lipid droplet interactions following Myls22 treatment compared to control cells (Figure 10P). Similarly, mitochondria–lysosome interactions were significantly enhanced upon Myls22 treatment, whereas Miclixin exhibited distinct or non‐significant effects depending on the interaction type analyzed (Figure 10Q). Finally, we assessed higher‐order organelle organization by quantifying three‐way mitochondria–lipid droplet–lysosome interactions. In contrast to the robust increases observed in pairwise interactions, three‐way interactions did not exhibit a significant increase under mitochondrial inner membrane perturbation (Figure 10R), indicating that enhanced pairwise organelle contacts do not necessarily translate into stabilization of higher‐order triorganelle assemblies.

### 3.14 Genetic Disruption of OPA1 and MICOS Components Impairs Mitochondrial Respiration

#### 3.14.1 Mouse Kidney Samples

**FIGURE 11:**
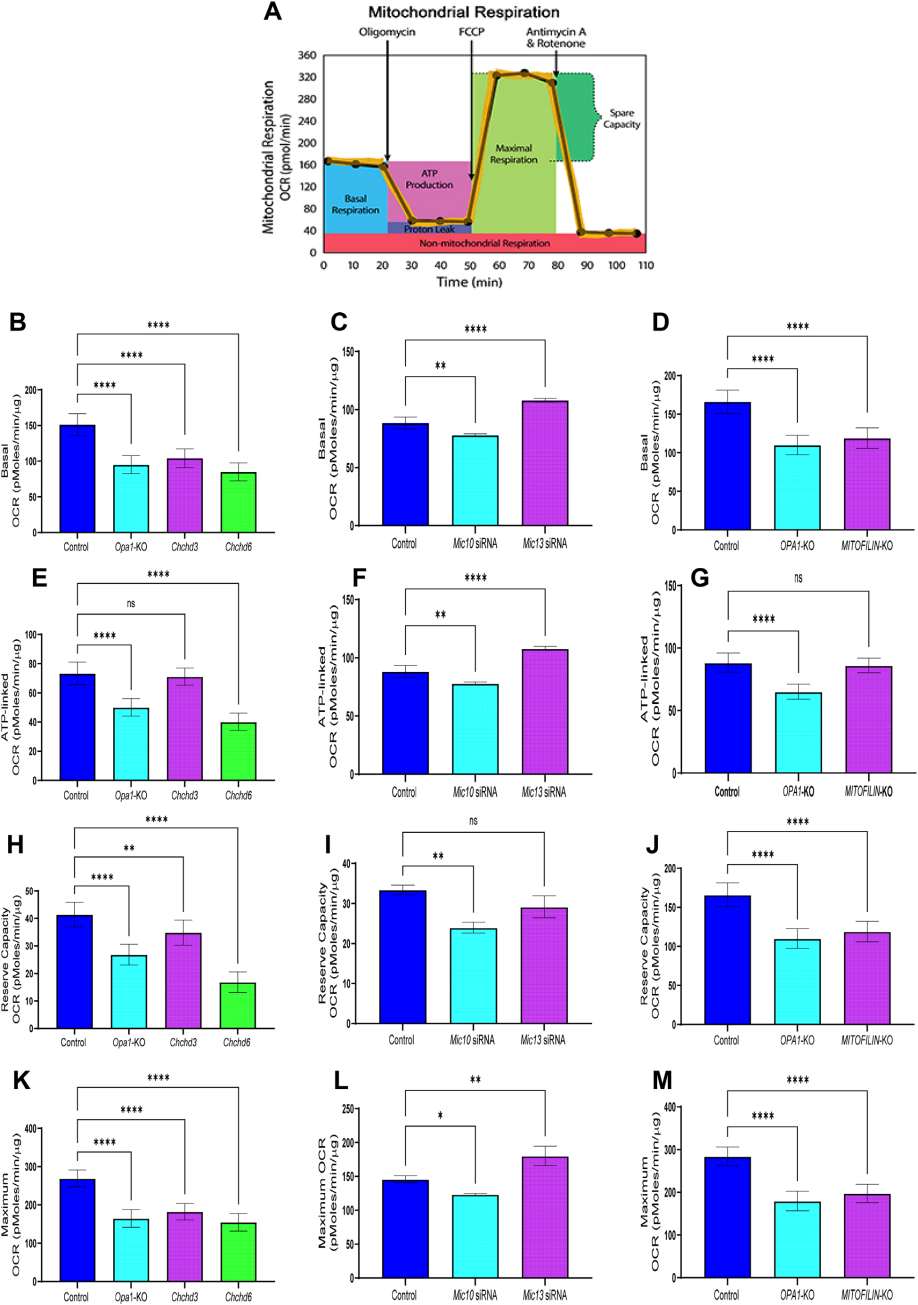
Genetic ablation of *Opa1* and siRNA‐mediated knockdown of MICOS components impair mitochondrial respiration. (A) Schematic representation of the Agilent Seahorse XF Cell Mito Stress Test, illustrating changes in oxygen consumption rate (OCR) following sequential injection of oligomycin, FCCP, and antimycin A/rotenone, and the derived mitochondrial respiratory parameters, including basal respiration, ATP‐linked respiration, maximal respiration, and reserve capacity. (B–M) Mitochondrial respiratory parameters measured using the Seahorse XF Cell Mito Stress Test. Panels are arranged vertically by experimental system and genetic perturbation. OCR values are normalized as indicated and presented as mean ± SEM. Each bar represents independent biological replicates. Statistical significance was determined using appropriate multiple‐comparison analyses as indicated. Significance is denoted as ns (not significant), *p* ≤ 0.05, *p* ≤ 0.01, and **p ≤ 0.0001. Mouse‐derived data (B, E, H, K) Basal OCR (B), ATP‐linked OCR (E), maximal OCR (H), and reserve capacity OCR (K) were measured in mouse samples from control, *Opa1* knockout (KO), *Chchd3* siRNA–mediated knockdown (KD), and *Chchd6* siRNA–mediated knockdown (KD) groups. (C, F, I, L) Basal OCR (C), ATP‐linked OCR (F), maximal OCR (I), and reserve capacity OCR (L) were measured in mouse samples treated with control siRNA, *Mic10* siRNA, or *Mic13* siRNA. Cultured cell data (D, G, J, M) Basal OCR (D), ATP‐linked OCR (G), maximal OCR (J), and reserve capacity OCR (M) were measured in HEK293 cells under control conditions or following genetic ablation of OPA1 or MITOFILIN, as indicated.

To assess the impact of MICOS components and OPA1 on mitochondrial respiratory function in vivo, mitochondrial respiration was measured in mouse samples using the Seahorse XF Cell Mito Stress Test (Figure 11A). Basal oxygen consumption rate (OCR) was significantly reduced in samples from *Opa1* knockout (KO), *Chchd3* knockdown (KD), and *Chchd6* KD mice compared with controls (Figure 11B). Analysis of ATP‐linked respiration revealed a significant decrease in *Opa1* KO and *Chchd6* KD samples, whereas *Chchd3* KD samples did not show a significant reduction relative to controls (Figure 11E). Maximal OCR was significantly reduced across *Opa1* KO, *Chchd3* KD, and *Chchd6* KD groups (Figure 11H), accompanied by a corresponding decrease in reserve respiratory capacity in these same conditions (Figure 11K). In parallel experiments targeting additional MICOS subunits, siRNA‐mediated knockdown of *Mic10* or *Mic13* resulted in a significant reduction in basal OCR compared with control siRNA–treated samples (Figure 11C). ATP‐linked respiration was modestly reduced following *Mic10* knockdown but not *Mic13* knockdown (Figure 11F). Maximal OCR and reserve capacity were both significantly reduced in *Mic10*‐ and *Mic13*‐depleted samples relative to controls (Figure 11I,L).

#### 3.14.2 HEK293 Cell Culture Model

To determine whether MICOS‐dependent respiratory defects are recapitulated in a cultured cell system, mitochondrial respiration was examined in HEK293 cells following genetic ablation of *OPA1* or Mitofilin. Basal OCR was significantly reduced in both *OPA1* KO and Mitofilin KO cells compared with control cells (Figure 11D). ATP‐linked OCR was significantly decreased in *OPA1* KO cells but did not differ significantly between Mitofilin KO and control cells (Figure 11G). Maximal OCR was significantly reduced in both *OPA1* KO and Mitofilin KO cells relative to controls (Figure 11J). Similarly, reserve respiratory capacity was significantly diminished in both genetic perturbations compared with control cells (Figure 11M).

Across both mouse‐derived samples and HEK293 cells, genetic disruption of OPA1 and multiple MICOS components consistently reduced basal and maximal mitochondrial respiration and diminished reserve respiratory capacity. However, ATP‐linked respiration exhibited perturbation‐specific effects that differed between individual MICOS components and between in vivo and in vitro systems.

### 3.15 MICOS Core Components MIC60 and CHCHD6 Are Required for Mitochondrial Ca^2+^ Uptake, Retention, and Redox Homeostasis

**FIGURE 12:**
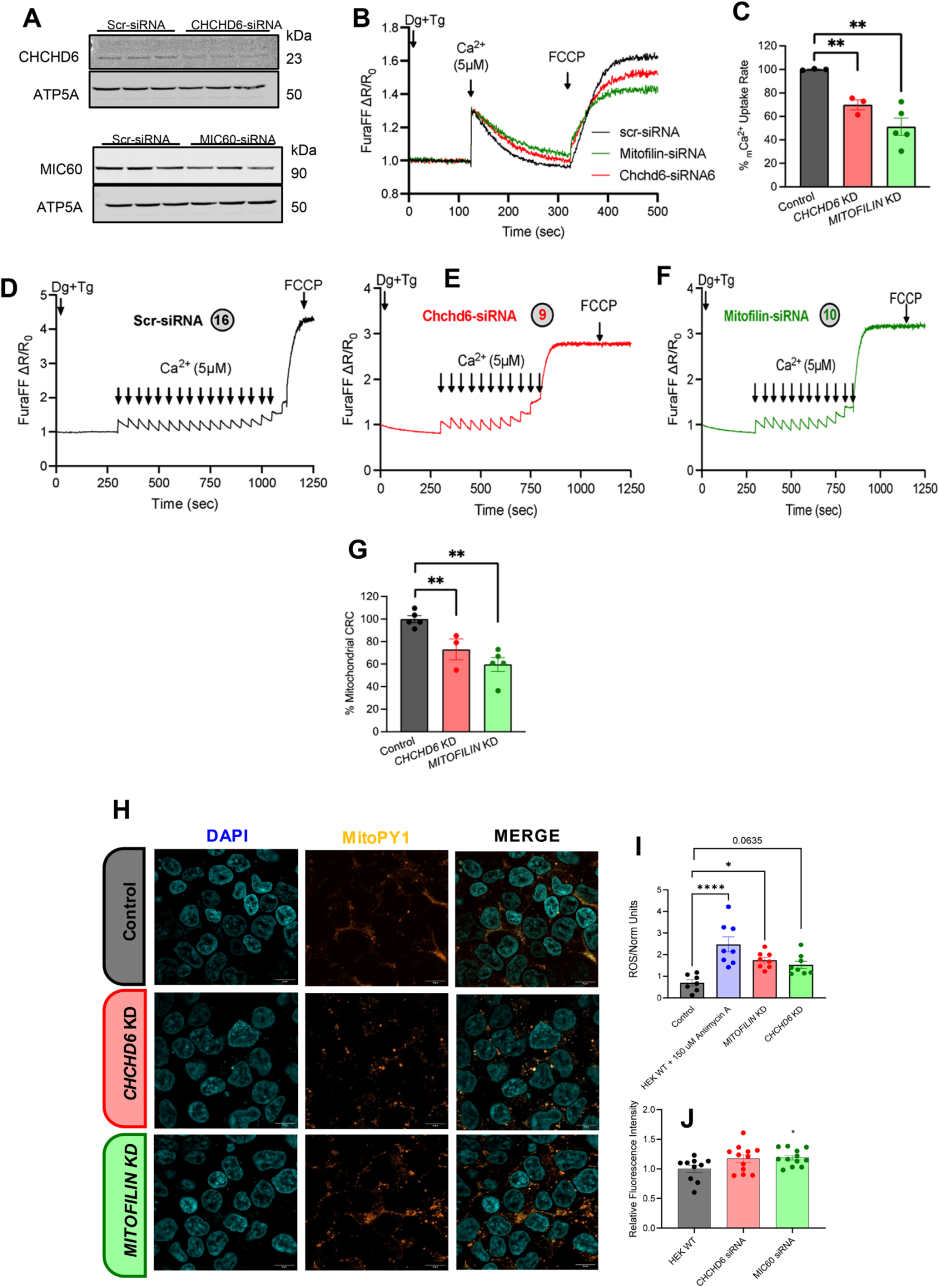
MICOS depletion disrupts mitochondrial calcium handling and redox balance in HEK293 cells. (A) Representative real‐time traces of mitochondrial Ca^2+^ (mCa^2+^) uptake in permeabilized HEK293 cells transfected with scramble siRNA (scr‐siRNA), MIC60‐siRNA, or CHCHD6‐siRNA. Traces were recorded using the ratiometric calcium indicator FuraFF, revealing altered mitochondrial calcium‐uptake kinetics upon MICOS depletion. Digitonin (Dg) and thapsigargin (Tg) were added as indicated to permeabilize the plasma membrane and inhibit endoplasmic reticulum Ca^2+^ uptake, respectively, followed by a Ca^2+^ pulse (5 μM) and FCCP addition to collapse the mitochondrial membrane potential. (B) Immunoblot validation of siRNA‐mediated knockdown efficiency in HEK293 cells. The top panel shows CHCHD6 protein levels following CHCHD6‐siRNA transfection; the bottom panel shows MIC60 protein levels following MIC60‐siRNA transfection. ATP5A was used as a mitochondrial loading control. (C–E) Representative mitochondrial calcium retention capacity (CRC) traces in permeabilized HEK293 cells transfected with scr‐siRNA (C), CHCHD6‐siRNA (D), or MIC60‐siRNA (E). Sequential Ca^2+^ boluses (5 μM each; indicated by arrows) were administered until mitochondrial permeability transition pore opening, as evidenced by a sudden increase in cytosolic Ca^2+^. (F) Quantification of mitochondrial CRC calculated from traces shown in (C–E), expressed as a percentage relative to scr‐siRNA control, demonstrating reduced calcium buffering capacity following MICOS component depletion. (G) Quantitative analysis of mitochondrial Ca^2+^ uptake rates derived from traces in (A), expressed as percentage change relative to control cells, revealing significant impairment upon MIC60 or CHCHD6 knockdown. Statistical analysis (panels A–G). For mitochondrial Ca^2+^ uptake kinetics and calcium retention capacity (CRC) analyses (A–G), representative traces are shown. Quantitative data in panels (F) and (G) were derived from independent biological replicates and are presented as mean ± SEM. Statistical significance was assessed using an unpaired two‐tailed Student's *t*‐test or one‐way ANOVA, as appropriate, with significance indicated as **p ≤ 0.01. (H) Representative fluorescence microscopy images of permeabilized HEK293 cells stained with DAPI (nuclei, blue) and the mitochondrial hydrogen peroxide sensor MitoPY1 (5 μM, 45 min at 37°C), following transfection with scr‐siRNA (control), MIC60‐siRNA (Mitofilin KD), or CHCHD6‐siRNA (CHCHD6 KD). Images were acquired at 60× magnification; merged channels are shown. (I) Quantification of mitochondrial ROS levels based on MitoPY1 fluorescence intensity from images shown in (H). (J) Relative MitoPY1 fluorescence emission intensity measured at 530 nm, normalized to control conditions, indicating altered mitochondrial redox status following MICOS depletion. Statistical analysis (panels H–J). For mitochondrial ROS measurements and MitoPY1 fluorescence quantification (H–J), data are presented as mean ± SEM, with each data point representing an independent biological replicate. Statistical significance was assessed using one‐way ANOVA followed by Dunnett's multiple‐comparisons test. Significance levels are indicated as *p ≤ 0.05, **p ≤ 0.01, ***p ≤ 0.001, ****p ≤ 0.0001; ns, not significant.

Mitochondrial Ca^2+^ handling is tightly linked to cristae architecture and inner membrane organization. To determine whether MICOS core components contribute directly to mitochondrial Ca^2+^ homeostasis, we examined mitochondrial Ca^2+^ uptake and retention in permeabilized HEK293 cells following siRNA‐mediated depletion of MIC60 or CHCHD6. Efficient knockdown of both MICOS components was confirmed by immunoblotting, with ATP5A serving as a mitochondrial loading control (Figure 12A). Real‐time measurements of mitochondrial Ca^2+^ uptake using the ratiometric indicator FuraFF revealed marked alterations in Ca^2+^ uptake kinetics upon MICOS depletion. Compared with scramble siRNA–treated cells, both MIC60‐ and CHCHD6‐knockdown cells exhibited reduced mitochondrial Ca^2+^ uptake following a defined Ca^2+^ pulse, despite comparable experimental conditions and intact mitochondrial membrane potential prior to FCCP addition (Figure 12B). Quantitative analysis confirmed a significant reduction in Ca^2+^ uptake rates in both knockdown conditions relative to control cells (Figure 12C), indicating that MICOS integrity is required for efficient mitochondrial Ca^2+^ entry.

We next assessed whether impaired Ca^2+^ uptake was accompanied by altered mitochondrial Ca^2+^ buffering capacity. Sequential Ca^2+^ bolus experiments demonstrated a pronounced reduction in mitochondrial calcium retention capacity (CRC) in MIC60‐ and CHCHD6‐knockdown cells compared with controls (Figure 12D–F). In both knockdown conditions, mitochondria underwent permeability transition pore opening after fewer Ca^2+^ additions, indicating increased susceptibility to Ca^2+^‐induced mitochondrial dysfunction. Quantification of CRC revealed a significant decrease in Ca^2+^ retention in MIC60‐ and CHCHD6‐depleted cells relative to scramble siRNA‐treated cells (Figure 12G).

Given the established coupling between mitochondrial Ca^2+^ dysregulation and oxidative stress, we next examined mitochondrial redox status following MICOS depletion. To assess mitochondrial redox status following MICOS depletion, mitochondrial hydrogen peroxide levels were measured in permeabilized HEK293 cells using the mitochondria‐targeted H_2_O_2_ sensor MitoPY1. Representative fluorescence images revealed increased MitoPY1 signal intensity in cells transfected with MIC60‐siRNA (Mitofilin KD) or CHCHD6‐siRNA compared with scramble siRNA‐treated controls (Figure 12H). Quantitative analysis of MitoPY1 fluorescence from microscopy images demonstrated a significant increase in mitochondrial ROS levels in both MIC60‐ and CHCHD6‐depleted cells relative to control cells (Figure 12I). Antimycin A treatment served as a positive control, producing a robust increase in mitochondrial ROS signal. Consistent with microscopy‐based measurements, quantification of MitoPY1 fluorescence emission intensity at 530 nm revealed a significant elevation in mitochondrial ROS levels following MIC60 or CHCHD6 knockdown compared with control conditions (Figure 12J).

Together, these data demonstrate that the MICOS core components MIC60 and CHCHD6 are essential for maintaining physiological mitochondrial Ca^2+^ uptake and retention. Disruption of MICOS integrity compromises mitochondrial Ca^2+^ handling, promotes early opening, and is associated with elevated mitochondrial oxidative stress, highlighting a critical role for cristae organization in safeguarding mitochondrial Ca^2+^ homeostasis.

## 4 Discussion

### 4.1 Structural Analysis

In the past, numerous studies have examined kidneys across various aging or disease states using TEM, which provides high‐resolution 2D images (Cui et al. 2012; Bárcena et al. 2010; O'Toole et al. 2010). While TEM is a useful technique for understanding changes in cristae integrity, it cannot accurately capture many structural details of mitochondria, such as the diverse structures they may adopt under cellular conditions (Peng et al. 2018). We found that TEM area measurements directly contradict our findings of decreased mitochondrial volume with aging. Here, we utilized SBF‐SEM to perform 3D reconstruction of the aged mouse kidney, which revealed many novel phenotypes and mitochondrial volume loss that were not otherwise captured by MRI or TEM. Using 3D reconstruction, we previously examined aged skeletal muscle in mice (Vue et al. 2022). We observed increased mitochondrial fragmentation accompanied by a compensatory increase in its structural complexity. In aging murine kidneys, mitochondrial complexity did not undergo significant changes. We noted a high rate of diverse or unique mitochondrial shapes, which may, in turn, have functional implications (Jenkins et al. 2024; Glancy et al. 2020). In this study, we found a large amount of variation in mitochondrial shape in both 3‐month and 2‐year‐old cohorts, which exhibit a mix of elongated, compact, large volume, small volume, nanotunnels, donut‐shaped, and branching structural phenotypes. While our previous studies in skeletal and cardiac tissue showed a dominant phenotype across aging, typically characterized by fragmentation, mitochondria in the kidney exhibit diverse shapes, and 3‐month and 2‐year samples do not show markedly different phenotypes.

One notable phenotype we observed is the presence of mitochondria in the form of a donut. We found that the 3‐month samples displayed many branched forms of mitochondria with high complexity, and within them, donut‐like structures formed. Past research utilizing 3D reconstruction in the aged brain of monkeys found a high rate of the donut mitochondria phenotype in the aged cohort, which had impaired memory function (Hara et al. 2014). Even beyond age, samples with mitochondrial donuts, which resulted in smaller synapses, had worse memory than older cohorts with normal mitochondria (Hara et al. 2014). While it is established that mitochondria donut are a hallmark of mitochondrial dysfunction (Picard and McEwen 2014), interestingly, they seem to be differentially expressed in tissue types and correlate with each tissue's functions. It has been suggested that their increased surface area relative to volume allows them to maintain more organelle contacts at the cost of lower ATP production (Glancy et al. 2020). It has also been found that, unlike swollen mitochondria, donut mitochondria maintain more of their internal structure and, potentially as a result, are not the target of mitophagy (Zhou, Long, et al. 2020). Since our analysis shows that more donuts occur in the younger samples, these may represent a positive phenotype in some cases. Therefore, the exact roles of donut mitochondria still remain unclear and may extend beyond what has been previously hypothesized.

Beyond changes in relative bioenergetics between mitochondrial shapes, their roles in calcium homeostasis and other biomolecular pathways deserve further research. For instance, past studies have also suggested that decreasing the activity of the Akt pathway, which is upstream from the mTORC pathway, may be a mechanism to restore autophagy, clear out defective mitochondria, and restore biogenetics (Jankauskas et al. 2018). However, given that other studies have shown that donut mitochondria may not be subject to autophagic clearing of mitochondria (Zhou, Long, et al. 2020), suggesting a potential reason for different relative rates of mitochondrial donut. Additionally, research on mitochondrial aging in the kidney has found that mtDNA is more error‐prone across aging, with up to a 5‐fold increase in the number of point mutations and deletions (Jankauskas et al. 2018), which may also be responsible for the alterations in mitochondrial structure as observed. Thus, the exact molecular underpinnings of these various shapes warrant further research.

Finally, we also found differences in mitochondrial structure across locations. Using 3D reconstruction, we found that in all aging kidney samples, mitochondria are round and located next to the nucleus. The further away from the nucleus, the more unique mitochondrial structures are present, such as those with a large volume (increased mitochondrial function capacity), small volume, elongated shape (relatively greater surface area facilitates interaction with the surrounding environment), compact structure, nano tunnels, and donut‐shaped appearance (increased surface area for interaction) are present (Jenkins et al. 2024). The change in shape likely arises from mitochondrial stress (Glancy et al. 2020; Long et al. 2015). Whereas areas that are not undergoing stress likely present mitochondria that are typical and elongated. Therefore, different areas of the kidney may undergo stress, potentially linked to stress from filtration, while others are less susceptible to stress as aging progresses. In a previous study, we found that mitochondria in cardiac tissue retain their morphology (Vue, Neikirk, et al. 2023). Therefore, a similar mechanism may exist that helps retain morphology for intracellular regions, such as perinuclear kidney mitochondria. Notably, the kidney houses at least 16 types of epithelial cells (Balzer et al. 2022) and has distinct regions, including the cortex, medulla, and renal sections, which serve different functions (Agarwal et al. 2013). However, our study did not permit the differentiation of these separate regions. Thus, future studies may consider using methods such as SDS‐PAGE to further differentiate kidney samples (Rockfield et al. 2024). Thus, future studies may further explore this by developing methods to separate kidney epithelial and globular areas using SBF‐SEM and examining whether mitochondrial differences are region‐ or area‐dependent across aging. Additionally, we were unable to perform sex comparisons, which may also reveal further heterogeneity across regions and between sexes.

### 4.2 Structural–Metabolic Coupling Drives Redox Imbalance in the Aging Kidney

Across aging, we observed coordinated structural and metabolic remodeling consistent with oxidative stress arising, in part, from disruption of the MICOS complex. Oxidative stress activates poly(ADP‐ribose) polymerase (PARP) as a DNA repair mechanism, which consumes nicotinamide adenine dinucleotide (NAD^+^), contributing to age‐associated NAD^+^ decline in both males and females (Massudi et al. 2012). Relevantly, extracellular NAD^+^ can trigger a cAMP‐dependent pathway that promotes Ca^2+^ influx and generation of superoxide and nitric oxide (Bruzzone et al. 2006). Reduced NAD^+^ availability has been implicated in mitochondrial dysfunction and diabetic kidney injury (Yan 2021), and studies using nicotinamide riboside to boost NAD^+^ levels suggest that simply restoring NAD^+^ pools may not fully rescue interorganelle communication or cristae dynamics (Lauritzen et al. 2015). Together, these findings support the possibility that MICOS‐dependent structural deterioration contributes to a redox environment that is not readily corrected by NAD^+^ repletion alone.

Our metabolic profiling revealed broad perturbations in amino acid, nucleotide, and redox metabolism in aged kidneys. We observed significant declines in methionine, valine, threonine, leucine, isoleucine, and glycine, highlighting disruption of pathways central to mitochondrial one‐carbon metabolism and branched‐chain amino acid utilization. Glycine, serine, and threonine metabolism—closely linked to the mitochondrial glycine cleavage system (GCS)—feed into purine biosynthesis and redox balance. Consistent with this, we detected marked reductions in purine intermediates, including inosine monophosphate (IMP), ribose‐phosphate, and related nucleotide metabolites. Because the Pentose Phosphate Pathway (PPP) supplies ribose‐5‐phosphate and NADPH, impairment of this pathway may simultaneously compromise nucleotide synthesis and antioxidant capacity (Ilter et al. 2023). PPP‐derived NADPH is essential for glutathione (GSH) regeneration, and depletion of nucleotides such as xanthine and ribose promotes genomic instability (Bester et al. 2011).

Interestingly, despite upstream perturbations in nucleotide biosynthesis, total adenylate pools (AMP, ADP, and ATP) were maintained in aged kidneys. This preservation of steady‐state ATP suggests that energetic homeostasis may be buffered through compensatory mechanisms, even as redox and biosynthetic pathways are significantly remodeled. One interpretation is that aging kidneys prioritize ATP maintenance at the expense of redox flexibility and anabolic capacity. Alternatively, these findings may reflect a temporal hierarchy in which redox imbalance and nucleotide depletion precede overt energetic collapse.

Consistent with prior literature on dysregulated NAD^+^ metabolism in aging kidneys (McReynolds et al. 2021), we observed significant declines in NAD^+^, NADP, and NAM, accompanied by accumulation of NADH (Figure 6H–K). This supports a redox shift hypothesis, wherein NAD^+^ is increasingly reduced to NADH without efficient re‐oxidation (McReynolds et al. 2020). Such an imbalance is consistent with impaired electron transport chain (ETC) activity, particularly in the context of altered cristae architecture. Further reinforcing disruption of oxidative metabolism, we detected an age‐dependent decline in FAD (Figure 6L). Similar to NAD(H), FAD functions as a critical electron carrier in oxidative phosphorylation, the TCA cycle, fatty acid β‐oxidation, and ETC complex II activity. Together, reductions in NAD^+^, NADP, and FAD, along with NADH accumulation, point to impaired oxidative capacity and a shift toward reductive stress rather than simple ATP depletion.

Our lipidomic profiling revealed substantial age‐associated remodeling of mitochondrial membrane lipids. TGOs and TGs serve as fatty acid storage pools and substrates for mitochondrial β‐oxidation (Mapuskar et al. 2023; Console et al. 2020), and their dysregulation may limit acetyl‐CoA generation. Sterols contribute to mitochondrial membrane integrity and fluidity (Zheng Koh and Saheki 2021; Tian et al. 2018), aligning with our structural observations of altered mitochondrial morphology. Of particular relevance, cardiolipins (DLCL and CL), phospholipids uniquely enriched in the inner mitochondrial membrane, are essential for cristae architecture and stabilization of respiratory chain supercomplexes (Paradies et al. 2019; Chen et al. 2018). Age‐associated alterations in cardiolipin abundance and composition likely compromise ETC organization and electron flux. Such structural perturbations provide a plausible mechanistic link between membrane remodeling and the observed redox imbalance, including NADH accumulation and FAD depletion.

Additional lipid classes, including NAEs, LPIs, and Hex2Cer, also exhibited age‐dependent changes. While NAEs are recognized mediators of endocannabinoid signaling (Tsuboi et al. 2018; Boachie et al. 2023) and LPIs function in signaling pathways, their roles in kidney mitochondrial biology remain incompletely defined. Hex2Cer contributes to mitochondrial membrane composition (Peng et al. 2018; Schenkel and Bakovic 2014) and may influence membrane curvature and organelle dynamics. Together, these lipidomic changes support a model in which age‐dependent membrane remodeling destabilizes mitochondrial structure, impairs respiratory efficiency, and reinforces redox and metabolic rewiring.

Importantly, these metabolomic and lipidomic measurements were performed in whole‐kidney tissue. While our structural analyses were specific to tubular cells, bulk tissue profiling may mask compartment‐ and cell‐type–specific metabolic alterations, particularly within mitochondrial ATP pools. Therefore, although our data strongly associate mitochondrial structural remodeling with redox imbalance and metabolic reprogramming, additional compartment‐resolved studies will be required to establish causality and define whether mitochondrial‐localized ATP production is preserved despite systemic buffering. Collectively, our findings support a model in which age‐associated disruption of MICOS‐dependent mitochondrial architecture initiates a cascade of membrane remodeling, redox imbalance, impaired nucleotide biosynthesis, and compensatory energetic buffering. Rather than immediate ATP failure, aging kidneys exhibit a state of redox‐constrained metabolism that may predispose them to injury under additional stress.

### 4.3 The MICOS Complex as a Master Regulator

With respect to AKI and CKD, mitochondria are known to play a significant role in the pathophysiology of these diseases (Jiang et al. 2020). Mitochondrial dynamics are complex, and observing key regulators of mitochondrial form and function may explain the changes that occur in kidney disease states. Key regulators of mitochondria include *OPA1* (mitochondrial fusion) and *DRP1* (mitochondrial fission), which may be responsible for the changes observed in the kidney. Past research has shown that in AKI, *OPA1* expression decreases and *DRP1* expression increases, suggesting mitochondrial fragmentation (Ishimoto and Inagi 2016). However, beyond models showing that decreased *DRP1* expression is not viable, mitochondrial fission is also important for maintaining various roles, including microtubule trafficking (Bowes and Gupta 2008). Therefore, this study sought to identify other targets and changes in mitochondrial structure beyond simple alterations in fusion and fission, which is often the extent of what TEM can survey. The MICOS complex is one such compelling target.

Aging in the kidneys is well‐established by us and others to cause interstitial fibrosis and oxidative stress (Chu et al. 2020; Gomes et al. 2009; Zhou et al. 2008; O'Sullivan et al. 2017). Our results suggested that age‐related loss of the MICOS complex leads to mitochondrial structural loss, generating oxidative stress and dysregulating calcium homeostasis. Since the MICOS complex forms across cristae junctions, the understanding of the interdependencies among MICOS complex proteins is still evolving. However, it is currently understood that some integral proteins, such as MIC60 (Mitofilin), regulate the expression of other proteins, including MIC10 and MIC19 (Rockfield et al. 2024). Similarly, MIC60/MIC19 (Mitofilin/Chchd3), unlike other MICOS complex proteins, assemble independently of cardiolipin, with MIC19 being responsible for regulating the distribution of subcomplexes (Friedman et al. 2015). Past studies of the MICOS complex in the kidney have been limited. However, they generally show that mitochondria‐rich regions, including the kidney, have a high rate of MIC60 and its isoforms. A deletion of *Mitofilin* results in a lethal disruption of the overall complex (Rockfield et al. 2024). This underscores the central role of Mitofilin, relative to other components of the MICOS complex, with functions that extend beyond cristae and mitochondrial dynamics to nucleoid distribution, suggesting roles in mtDNA synthesis (Li et al. 2016). This has been recapitulated by other studies, which show that Mitofilin depletion decreases mtDNA transcription, resulting in impaired bioenergetics in the kidney, as previously reviewed (Feng et al. 2019). Notably, we observed a marked age‐related decrease in Mitofilin compared with other components; however, Mitofilin also showed a less drastic mitochondrial phenotype when knocked out than other MICOS complex proteins. While the structural analysis of MICOS complex knockouts is limited to TEM, this underscores the importance of considering other roles of the MICOS complex beyond its well‐established, extensively reviewed function in cristae dynamics and biogenesis (Anand et al. 2021; Viana et al. 2021).

The role of the MICOS complex in disease states remains controversial. Loss of the MICOS complex has been shown to reduce cardiac ATP levels, thereby impairing tissue integrity (Birker et al. 2023). Studies in other tissues, such as the liver, have shown that *CHCHD3* depletion impairs MERCs, leading to fatty liver disease with *SLC25A46* involvement (Dong et al. 2024). As previously reviewed, the MICOS complex has thus been involved in neurodegenerative disorders, metabolic syndromes, cardiac dysfunctions, and muscle pathologies (Eramo et al. 2020). In the kidney, as previously reviewed, impairment of Mitofilin has been specifically implicated in the pathophysiology of mtDNA‐related renal diseases, diabetic kidney disease, kidney failure, and reperfusion (Feng et al. 2019). Interestingly, other studies have suggested a protective mechanism due to the loss of the MICOS complex. Loss of the MICOS complex, despite an aberrant cristae structure, is a protective factor against aging, as it exhibits an unexpected, pronounced lifespan extension in *Podospora anserina* (Warnsmann et al. 2022). Specifically, it has been suggested that Miro‐MIC60 interactions impair cellular respiration and cause oxidative stress, thereby preventing mitophagy and increasing susceptibility to Parkinson's disease and Friedreich's ataxia (Li et al. 2021). This underscores the need to better understand the impact of the MICOS complex loss.

Notably, unlike other studies that show Miro‐MIC60 interactions cause oxidative stress, we found that deletion of MICOS complex components Chchd6 and Mitofilin resulted in mitochondrial and cellular oxidative stress. As previously reviewed, oxidative stress has been observed following *Mitofilin* deletion in some tissues, such as the heart, but the interplay between the MICOS complex and oxidative stress remains poorly understood (Ilter et al. 2023). Notably, within the kidney, oxidative stress mediates age‐associated renal cell death and has been linked to numerous pathological conditions, as previously reviewed (Percy et al. 2005). Since the loss of the MICOS complex is well understood to impair bioenergetics and ATP production (Vue, Garza‐Lopez, et al. 2023; An et al. 2012), our findings suggest that the closely linked process of free radical generation is also strengthened. MICOS‐generated ROS may have various effects; for example, they can reduce NAD^+^, as observed in our aged tissue, leading to alterations in glycolysis, the TCA cycle, and oxidative phosphorylation, as previously reviewed (Yang and Sauve 2016). reviewed: changes in fuel availability alter TCA metabolite levels, with downstream effects on reduced mitochondrial calcium uptake and lowered matrix Ca^2+^ levels., in turn, decreases Ca^2+^‐dependent TCA cycle enzyme activity, including pyruvate dehydrogenase and α‐ketoglutarate dehydrogenase, and, in some cases, induces autophagy as a compensatory mechanism for changes in substrate availability (Tomar and Elrod 2020). Since we observed a concomitant decrease in mCa^2+^ uptake upon silencing of the MICOS complex, this suggests a vicious cycle in which ROS‐dependent NAD+ and calcium‐dependent TCA metabolites are lost due to MICOS; however, this pathway warrants further exploration. Alternatively, oxidative stress can cause mitochondrial permeability transition pore (mPTP) openings, which adaptively release excess ROS to maintain mitochondrial homeostasis; however, in pathological, persistent conditions, these pores can engage in destructive ROS‐dependent ROS release (Gottschalk et al. 2019; Tomar et al. 2023). While mPTP openings can be transient, calcium‐dependent lowering of the membrane potential can also cause permanent openings, which confer an increased risk of apoptotic pathways (Li et al. 2010), suggesting an alternative pathway through which a feedback loop may arise due to ROS generation and calcium dysregulation following the silencing of proteins involved in the MICOS complex.

In murine renal tubular epithelial cells, an MCU‐dependent increase in mitochondrial calcium accumulation leads to oxidative stress and, ultimately, senescence (Xiong et al. 2024). This study examines the importance of further elucidating MICOS's role in senescence and the therapies that target this process. For example, a recent study found that diminished Glis1 expression in age‐related kidney aging models correlates with impaired mitochondrial quality control mechanisms. In contrast, increased Glis1 interaction with PGC‐1α helps maintain mitochondrial stability, suggesting *Glis1* as a potential therapeutic target for mitigating cell senescence and age‐related renal fibrosis (Xu et al. 2023). Furthermore, the role of the MICOS complex in regulating calcium highlights the importance of investigating other regulators of the mitochondrial Ca^2+^ uniporter (e.g., MICU1, MCU, EMRE), some of which have recently been shown to influence cristae morphology (Gottschalk et al. 2019). While MICU1 has increasingly been shown to have a role in cristae morphology, the interconnectedness of these proteins has not yet been studied in the context of the MICOS complex's downstream effectors (Gottschalk et al. 2019; Tomar et al. 2023).

Accumulating evidence links nicotinamide adenine dinucleotide phosphate reduced oxidase (NOX)‐driven oxidative stress to ER stress‐induced apoptosis and subsequent renal dysfunction (Li et al. 2010). Similarly, NOXs have been indicated to play a role in acute kidney injury by promoting oxidative stress (Li et al. 2017; Jeong et al. 2018). Beyond underscoring the therapeutic potential of NOXs, their interdependence with ER stress also highlights the importance of further studying MERCs. MERCs, which contact sites under 50 nm that can be caused by ER stress, have previously been associated with calcium signaling and lipid metabolism. However, recent research has further suggested a potential role in senescence (Ziegler et al. 2021). Here, we did not comprehensively study MERCs, which are known to be implicated in mitochondrial calcium homeostasis (Carreras‐Sureda et al. 2019). However, a qualitative analysis did show that wrappER forms principally in young samples. Past studies have shown that the rough endoplasmic reticulum may curve to closely wrap around the mitochondria and maintain lipid homeostasis, a phenomenon termed wrappER (Anastasia et al. 2021). Thus, the lipidomic shifts we observed with aging may be caused by deficient lipid flux and impaired cristae structure, without wrappER. This compartment, which performs numerous functions, including fatty acid secretion, may serve as an organelle linking mitochondria and peroxisomes to regulate overall lipid balance (Ilacqua et al. 2021). Given that calcium homeostasis dysfunction is a potential avenue for kidney disease (Ishimoto and Inagi 2016), it remains important to consider in the future how calcium homeostasis is impacted across aging through MERC modulation. Furthermore, these qualitative findings must be confirmed by an extensive quantitative study, similar to the one we performed for mitochondria.

## 5 Conclusion

Our results demonstrate that the aging of murine kidney tissue is associated with cristae disarray and impaired mitochondrial structure, resulting in reduced organelle volume. This occurs alongside widespread metabolic and lipidomic shifts, as well as increased fibrosis and oxidative stress, which collectively reduce oxidative capacity and increase the risk of age‐related disease states, including CKD and AKI (Gyurászová et al. 2020). We further found that the MICOS complex is lost with kidney aging, in the absence of changes in other standard regulators of mitochondria and cristae morphology. While the age‐dependent loss of the MICOS complex likely accounts for the loss of cristae architecture, silencing of MICOS components in HEK cells confers a structure similar to that of aged tissue. The MICOS complex silencing further causes oxidative stress. It is plausible that these changes result in a vicious cycle: MICOS loss drives oxidative stress, leading to calcium‐dependent TCA dysregulation and NAD^+^ dysregulation, which in turn drive more oxidative stress and mtDNA loss, leading to a reduction in MICOS complex transcripts and a subsequent reduction in mitochondrial function, producing more oxidative stress byproducts, and ultimately leading to age‐dependent disease states. Loss of the MICOS complex alters mitochondrial morphology, leading to distinct changes in mitochondrial structure. We frequently observed circular or ring‐shaped mitochondria—commonly referred to as “donut” mitochondria—which are often associated with continuous mitochondrial constriction. The size of these mitochondrial donuts appears critical for modulating bending energy, a key biophysical barrier to their formation. This energy barrier may be counterbalanced by stress‐inducible mitochondrial osmotic pressure. For instance, rotenone (a Complex I inhibitor) and antimycin A (a Complex III inhibitor) are known to induce ROS, triggering a transition from tubular to donut‐ or blob‐shaped mitochondria. These ROS‐associated donut formations are typically transient and reversible, suggesting an adaptive response to oxidative stress (Jenkins et al. 2024; Glancy et al. 2020; Ahmad et al. 2013; Long et al. 2015; Scudese et al. 2025). In our study, we also identified numerous compact mitochondria, further indicating disrupted mitochondrial dynamics. These morphological changes were consistently observed when comparing 3‐month‐old and 2‐year‐old tissue, with greater fragmentation in the aged samples. Transmission electron microscopy (TEM) confirmed these structural transitions. Together, these findings support a model in which the MICOS complex, together with OPA1, is essential for regulating mitochondrial dynamics and inner‐membrane remodeling. This regulation maintains mitochondrial membrane potential, calcium buffering capacity, and cristae architecture—key components of mitochondrial function that are critical for proper kidney physiology. Interestingly, the ring‐ and donut‐like mitochondrial structures observed following MICOS complex loss resemble those reported in our recent study of MFN‐2–mediated mitochondrial remodeling in aged human skeletal muscle (Scudese et al. 2025). In both contexts, we observed a consistent emergence of toroidal and compact mitochondrial forms, suggesting that despite acting on different structural components—the MICOS complex at the cristae junction and MFN‐2 at the outer membrane tethering/fusion sites—both proteins influence mitochondrial architecture in a convergent manner. The buffering of mitochondrial membrane potential and the control of bending energy may represent shared mechanisms underlying these morphological transitions. Moreover, as we reported in Scudese et al., MFN‐2 loss was associated with changes in mitochondrial volume, shape complexity, and network fragmentation, features also observed with MICOS deficiency. These findings suggest that inner and outer membrane remodeling pathways may be mechanistically linked through a shared biophysical constraint, such as osmotic stress or impaired fusion, that drives adaptive mitochondrial shaping under aging or stress conditions.

## Author Contributions

**Conceptualization:** Antentor Hinton Jr., Melanie R. McReynolds. **Methodology:** Prasanna Katti, Praveena Prasad, Sepiso K. Masenga, Zer Vue, Prasanna Venkhatesh, Andrea G. Marshall, Benjamin Rodriguez, Han Le, Edgar Garza‐Lopez, Alexandria Murphy, Brenita Jenkins, Ashlesha Kadam, Jianqiang Shao, Amber Crabtree, Pamela Martin, Chantell Evans, Mark A. Phillips, David Hubert, Nelson Wandira, Okwute M. Ochayi, Dhanendra Tomar, Clintoria R. Williams, Jennifer Gaddy, Briar Tomeau, LaCara Bell, Taneisha Gillyard, Markis' Hamilton, Vineeta Sharma, Amadou Gaye, Elma Zaganjor, Olujimi A. Ajijola, Estevão Scudese, Tyne W. Miller Fleming, André Kinder, Chandravanu Dash, Anita M. Quintana, Bret C. Mobley, Julia D. Berry, Pooja Jadiya, Dao‐Fu Dai, Annet Kirabo, Oleg Kovtun, Jenny C. Schafer, Sean Schaffer, Renata Oliveira Pereira. **Investigation:** Zer Vue, Praveena Prasad, Sepiso K. Masenga, Prasanna Katti, Prasanna Venkhatesh, Andrea G. Marshall, Benjamin Rodriguez, Han Le, Mohd M khan and all middle authors listed above. **Formal Analysis:** Prasanna Katti, Zer Vue, Praveena Prasad, Sepiso K. Masenga, Prasanna Venkhatesh. **Visualization:** Prasanna Katti, Zer Vue, Praveena Prasad, Sepiso K. Masenga, Prasanna Venkhatesh. **Writing – original draft:** Prasanna Katti, Zer Vue, Praveena Prasad, Sepiso K. Masenga, Mohd M Khan. **Writing – review and editing:** Prasanna Venkhatesh, Prasanna Katti, Sepiso K. Masenga, Mohd, M khan. **Resources:** Antentor Hinton Jr., Melanie R. McReynolds. **Supervision:** Antentor Hinton Jr., Melanie R. McReynolds. **Project administration:** Antentor Hinton Jr., Melanie R. McReynolds. **Funding acquisition:** Antentor Hinton Jr., Melanie R. McReynolds.

## Funding

The BioVU project at VUMC is supported by numerous sources, including NIH‐funded Shared Instrumentation Grants S10OD017985, S10RR025141, and S10OD025092, and CTSA grants UL1TR002243, UL1TR000445, and UL1RR024975. Its contents are solely the responsibility of the authors and do not necessarily represent official views of the National Centre for Advancing Translational Sciences. Genomic data are also supported by investigator‐led projects, including U01HG004798, R01NS032830, RC2GM092618, P50GM115305, U01HG006378, U19HL065962, and R01HD074711, as well as additional funding sources listed at https://victr.vumc.org/biovu‐funding/. This project was funded by the National Institute of Health (NIH) NIDDK T‐32, number DK007563 entitled Multidisciplinary Training in Molecular Endocrinology to Z.V.; National Institute of Health (NIH) NIDDK T‐32, number DK007563 entitled Multidisciplinary Training in Molecular Endocrinology to A.C.; NSF MCB #2011577I to S.A.M.; The UNCF/Bristol‐Myers Squibb E.E. Just Faculty Fund, Career Award at the Scientific Interface (CASI Award) from Burroughs Welcome Fund (BWF) ID # 1021868.01, BWF Ad‐hoc Award, NIH Small Research Pilot Subaward to 5R25HL106365–12 from the National Institutes of Health PRIDE Program, DK020593, Vanderbilt Diabetes and Research Training Center for DRTC Alzheimer's Disease Pilot & Feasibility Program. CZI Science Diversity Leadership grant number 2022‐253529 from the Chan Zuckerberg Initiative DAF, an advised fund of Silicon Valley Community Foundation, to A.H.J., and National Institutes of Health grant HD090061 and the Department of Veterans Affairs Office of Research Award I01 BX005352 to J.G. Howard Hughes Medical Institute Hanna H. Gray Fellows Program Faculty Phase (Grant# GT15655 awarded to M.R.M); and Burroughs Wellcome Fund PDEP Transition to Faculty (Grant# 1022604 awarded to M.R.M). National Institutes of Health Grants: R21DK119879 (to C.R.W.) and R01DK‐133698 (to C.R.W.), American Heart Association Grant 16SDG27080009 (to C.R.W.) and by an American Society of Nephrology KidneyCure Transition to Independence Grant (to C.R.W.). Doris Duke Clinical Scientist Development Award grant 2021193, Burroughs Wellcome Fund grant 1021480, K23 HL156759, and R01 DK112262 (CNW). NIH Grants 2D43TW009744 (SKM), R21TW012635 (AK and SKM) and the American Heart Association Award Number 24IVPHA1297559 https://doi.org/10.58275/AHA.24IVPHA1297559.pc.gr.193866 (SKM). International federation for clinical chemistry (SKM). NIH Grants R01HL147818, R03HL155041, and R01HL144941 (A. Kirabo). NIH Grant R00DK120876 (D.T.), Harold S. Geneen Charitable Trust Awards Program (D.T.), Alzheimer's Association AARG‐NTF‐23‐1144888 (D.T.). NIH Grant R00AG065445 (P.J.), Alzheimer's Association 24AARG‐D‐1191292 (P.J.), Wake ADRC REC and Development grant P30AG072947 (P.J.). American Heart Association Grant 23POST1020344 (A.K.). NIH K01AG062757 to (M.T.S.) ANRF (Anusandhan National Research Foundation), ANRF/ECRG/2024/001042/LS, ANRF/IRG/2024/001777/LS. IISER Tirupati, NFSG. (P.K) Confocal microscopy, super‐resolution microscopy, and image processing were performed through the Vanderbilt Cell Imaging Shared Resource (CISR), supported by CA68485, DK58404, and EY08126. S10MH130456 funded the Nikon Spinning Disk Confocal SoRa microscope. Its contents are solely the responsibility of the authors and do not necessarily represent the official view of the NIH. The contents are solely the responsibility of the authors and do not necessarily represent the official view of the NIH. The funders had no role in study design, data collection, and analysis, decision to publish, or preparation of the manuscript. We would also like to acknowledge the Huck Institutes' Metabolomics Core Facility (RRID:SCR_023864) for use of the OE 240 LCMS and Drs. Imhoi Koo, Ashley Shay, and Sergei Koshkin for helpful discussions on sample preparation and analysis. The *All of Us* data analysis was supported by a grant (1 OT2 OD031932) from the National Institutes of Health to DDM. The *All of Us* Research Program is supported by the National Institutes of Health, Office of the Director, to participating organizations: Regional Medical Centers (1 OT2 OD026549; 1 OT2 OD026554; 1 OT2 OD026557; 1 OT2 OD026556; 1 OT2 OD026550; 1 OT2 OD 026552; 1 OT2 OD026553; 1 OT2 OD026548; 1 OT2 OD026551; 1 OT2 OD026555; IAA #: AOD 16037); Federally Qualified Health Centers (HHSN 263201600085 U); Data and Research Center (5 U2C OD023196); Biobank (1 U24 OD023121); The Participant Center (U24 OD023176); Participant Technology Systems Center (1 U24 OD023163); Communications and Engagement (3 OT2 OD023205; 3 OT2 OD023206); and Community Partners (1 OT2 OD025277; 3 OT2 OD025315; 1 OT2 OD025337; 1 OT2 OD025276).

## Conflicts of Interest

The authors declare no conflicts of interest.

## Supporting information


**Table S1:** Nominally significant genitourinary phecodes associated with CHCHD6 loss‐of‐function carrier status in the *All of Us* cohort.
**Table S2:** Nominally significant genitourinary phecodes associated with OPA1 loss‐of‐function carrier status in the *All of Us* cohort.

**Table S3:** Results from analysis comparing individual lipids between old and young kidney samples.
**Table S4:** Results for set enrichment analysis, lipid class and chain length, based on lipidomic differentiation between young and old kidney tissue.

**Figure S1:** Flowchart of CHCHD6 loss‐of‐function carrier cohort selection for covariate‐adjusted PheWAS analysis from the *All of Us* Research Program. Participants were selected from the *All of Us* Research Program using Controlled Tier Dataset version 8, including participants enrolled and consented through October 1, 2023. Among 633,248 participants aged ≥ 18 years at consent, participants without srWGS data, without linked EHR data, or with assigned sex at birth other than male or female or missing and declined responses were excluded. CHCHD6 rare predicted LoF carriers were defined using variants annotated as frameshift, splice‐acceptor, splice‐donor, or stop‐gained, with allele frequency ≤ 0.001. This yielded an initial eligible case–control cohort of 314,519 participants, including 148 CHCHD6 LoF carriers and 314,371 noncarriers. After derivation of regression covariates and removal of participants with missing covariate data, the final covariate‐complete analytic sample comprised 180,076 participants, including 86 CHCHD6 LoF carriers and 179,990 noncarriers. srWGS, short‐read whole‐genome sequencing; EHR, electronic health record; LoF, loss‐of‐function.
**Figure S2:** Flowchart of OPA1 loss‐of‐function carrier cohort selection for covariate‐adjusted PheWAS analysis from the *All of Us* Research Program. Participants were selected from the *All of Us* Research Program using Controlled Tier Dataset version 8, including participants enrolled and consented through October 1, 2023. Among 633,248 participants aged ≥ 18 years at consent, participants without srWGS data, without linked EHR data, or with assigned sex at birth other than male or female or missing and declined responses were excluded. OPA1 rare predicted LoF carriers were defined using variants annotated as frameshift, splice‐acceptor, splice‐donor, or stop‐gained, with allele frequency ≤ 0.001. This yielded an initial eligible case–control cohort of 314,519 participants, including 83 OPA1 LoF carriers and 314,436 noncarriers. After derivation of regression covariates and removal of participants with missing covariate data, the final covariate‐complete analytic sample comprised 180,076 participants, including 49 OPA1 LoF carriers and 180,027 noncarriers. srWGS, short‐read whole‐genome sequencing; EHR, electronic health record; LoF, loss‐of‐function.

## Data Availability

Sharing of software, models, algorithms, protocols, methods, and other useful materials and resources related to the manuscript will be available on a public repository upon publication.
