# Supplementary material for: The MICOS Complex Regulates Mitochondrial Structure and Oxidative Stress During Age‐Dependent Structural Deficits in the Kidney
Source: Aging Cell. 2026 May 11;25(5):e70534. doi: 10.1111/acel.70534 (PMC13160932; doi:10.1111/acel.70534)
Supplement: Supplementary file 1 — Table S1: Nominally significant genitourinary phecodes associated with CHCHD6 loss‐of‐function carrier status in the All of Us cohort. Table S2: Nominally significant genitourinary phecodes associated with OPA1 loss‐of‐function carrier status in the All of Us cohort. [file ACEL-25-e70534-s001.docx]

**Suppleentary Table 1. Nominally significant genitourinary phecodes associated with CHCHD6 loss-of-function carrier status in the *All of Us* cohort.** Each row represents a phecode-defined genitourinary phenotype nominally associated with CHCHD6 predicted loss-of-function carrier status in covariate-adjusted logistic regression. Carrier status was modeled as the independent variable of interest and phecode case status as the outcome, adjusting for age at last event, sex at birth, and the first five principal components. Cases and controls indicate the numbers of participants in the analytic cohort with and without the corresponding phecode. OR, odds ratio; CI, confidence interval.

| **Phecode** | **Phenotype** | **Cases** | **Controls** | **OR (95% CI)** | ***p*-value** |
| --- | --- | --- | --- | --- | --- |
| 597.8 | Urethral hypermobility/ISD | 246 | 179587 | 18.438 | 6.471e-05 |
| 580.12 | Non-proliferative glomerulonephritis | 257 | 179683 | 17.82 | 6.576e-05 |
| 593.1 | Gross hematuria | 3449 | 174549 | 4.461 | 2.612e-04 |
| 602.3 | Dysplasia of prostate | 60 | 67297 | 31.505 | 6.068e-04 |
| 618.2 | Uterine/Uterovaginal prolapse | 1576 | 110327 | 6.251 | 6.697e-04 |
| 618 | Genital prolapse | 4524 | 106292 | 3.941 | 1.244e-03 |
| 580.4 | Renal sclerosis, NOS | 499 | 178941 | 9.272 | 1.961e-03 |
| 587 | Kidney replaced by transplant | 1149 | 178632 | 6.128 | 2.210e-03 |
| 580.1 | Glomerulonephritis | 513 | 179228 | 8.61 | 2.736e-03 |
| 597 | Other disorders of urethra and urinary tract | 1091 | 177592 | 5.8 | 3.176e-03 |
| 618.1 | Prolapse of vaginal walls | 3026 | 108278 | 4.166 | 3.685e-03 |
| 580.32 | Nephritis and nephropathy with pathological lesion | 722 | 178576 | 6.164 | 1.126e-02 |
| 601.3 | Orchitis and epididymitis | 798 | 65880 | 5.371 | 2.204e-02 |
| 599.9 | Other abnormality of urination | 7242 | 166610 | 2.384 | 2.255e-02 |
| 599.6 | Oliguria and anuria | 247 | 179201 | 8.56 | 3.350e-02 |
| 593.2 | Microscopic hematuria | 1851 | 176466 | 3.472 | 3.620e-02 |
| 593 | Hematuria | 10928 | 161191 | 2.02 | 4.260e-02 |

**Supplemetary Table 2. Nominally significant genitourinary phecodes associated with OPA1 loss-of-function carrier status in the *All of Us* cohort.** Each row represents a phecode-defined genitourinary phenotype nominally associated with OPA1 predicted loss-of-function carrier status in covariate-adjusted logistic regression. Carrier status was modeled as the independent variable of interest and phecode case status as the outcome, adjusting for age at last event, sex at birth, and the first five principal components. Cases and controls indicate the numbers of participants in the analytic cohort with and without the corresponding phecode. OR, odds ratio; CI, confidence interval.

| **Phecode** | **Phenotype** | **Cases** | **Controls** | **OR (95% CI)** | ***p*-value** |
| --- | --- | --- | --- | --- | --- |
| 580.11 | Proliferative glomerulonephritis | 155 | 179792 | 24.044 | 1.747e-03 |
| 599.7 | Urethral discharge | 199 | 179512 | 18.452 | 6.169e-03 |
| 580.12 | Non-proliferative glomerulonephritis | 257 | 179683 | 14.95 | 7.774e-03 |
| 619.3 | Noninflammatory disorders of cervix | 1216 | 109216 | 5.427 | 2.047e-02 |
| 596.5 | Functional disorders of bladder | 4787 | 173104 | 3.376 | 2.152e-02 |
| 618.1 | Prolapse of vaginal walls | 3026 | 108278 | 3.931 | 2.727e-02 |
| 618 | Genital prolapse | 4524 | 106292 | 3.327 | 2.739e-02 |
| 618.2 | Uterine/Uterovaginal prolapse | 1576 | 110327 | 4.759 | 3.494e-02 |
| 614.53 | Cyst or abscess of Bartholin's gland | 414 | 111803 | 8.186 | 3.904e-02 |
| 610.4 | Benign neoplasm of breast | 1636 | 109401 | 4.304 | 4.617e-02 |
| 580.1 | Glomerulonephritis | 513 | 179228 | 7.52 | 4.649e-02 |
