# Supplementary material for: The MICOS Complex Regulates Mitochondrial Structure and Oxidative Stress During Age‐Dependent Structural Deficits in the Kidney
Source: Aging Cell. 2026 May 11;25(5):e70534. doi: 10.1111/acel.70534 (PMC13160932; doi:10.1111/acel.70534)
Supplement: Supplementary file 3 — Figure S1: Flowchart of CHCHD6 loss‐of‐function carrier cohort selection for covariate‐adjusted PheWAS analysis from the All of Us Research Program. Participants were selected from the All of Us Research Program using Controlled Tier Dataset version 8, including participants enrolled and consented through October 1, 2023. Among 633,248 participants aged ≥ 18 years at consent, participants without srWGS data, without linked EHR data, or with assigned sex at birth other than male or female or missing and declined responses were excluded. CHCHD6 rare predicted LoF carriers were defined using variants annotated as frameshift, splice‐acceptor, splice‐donor, or stop‐gained, with allele frequency ≤ 0.001. This yielded an initial eligible case–control cohort of 314,519 participants, including 148 CHCHD6 LoF carriers and 314,371 noncarriers. After derivation of regression covariates and removal of participants with missing covariate data, the final covariate‐complete analytic sample comprised 180,076 participants, including 86 CHCHD6 LoF carriers and 179,990 noncarriers. srWGS, short‐read whole‐genome sequencing; EHR, electronic health record; LoF, loss‐of‐function. Figure S2: Flowchart of OPA1 loss‐of‐function carrier cohort selection for covariate‐adjusted PheWAS analysis from the All of Us Research Program. Participants were selected from the All of Us Research Program using Controlled Tier Dataset version 8, including participants enrolled and consented through October 1, 2023. Among 633,248 participants aged ≥ 18 years at consent, participants without srWGS data, without linked EHR data, or with assigned sex at birth other than male or female or missing and declined responses were excluded. OPA1 rare predicted LoF carriers were defined using variants annotated as frameshift, splice‐acceptor, splice‐donor, or stop‐gained, with allele frequency ≤ 0.001. This yielded an initial eligible case–control cohort of 314,519 participants, including 83 OPA1 LoF carriers and 314,436 [file ACEL-25-e70534-s003.docx]

**
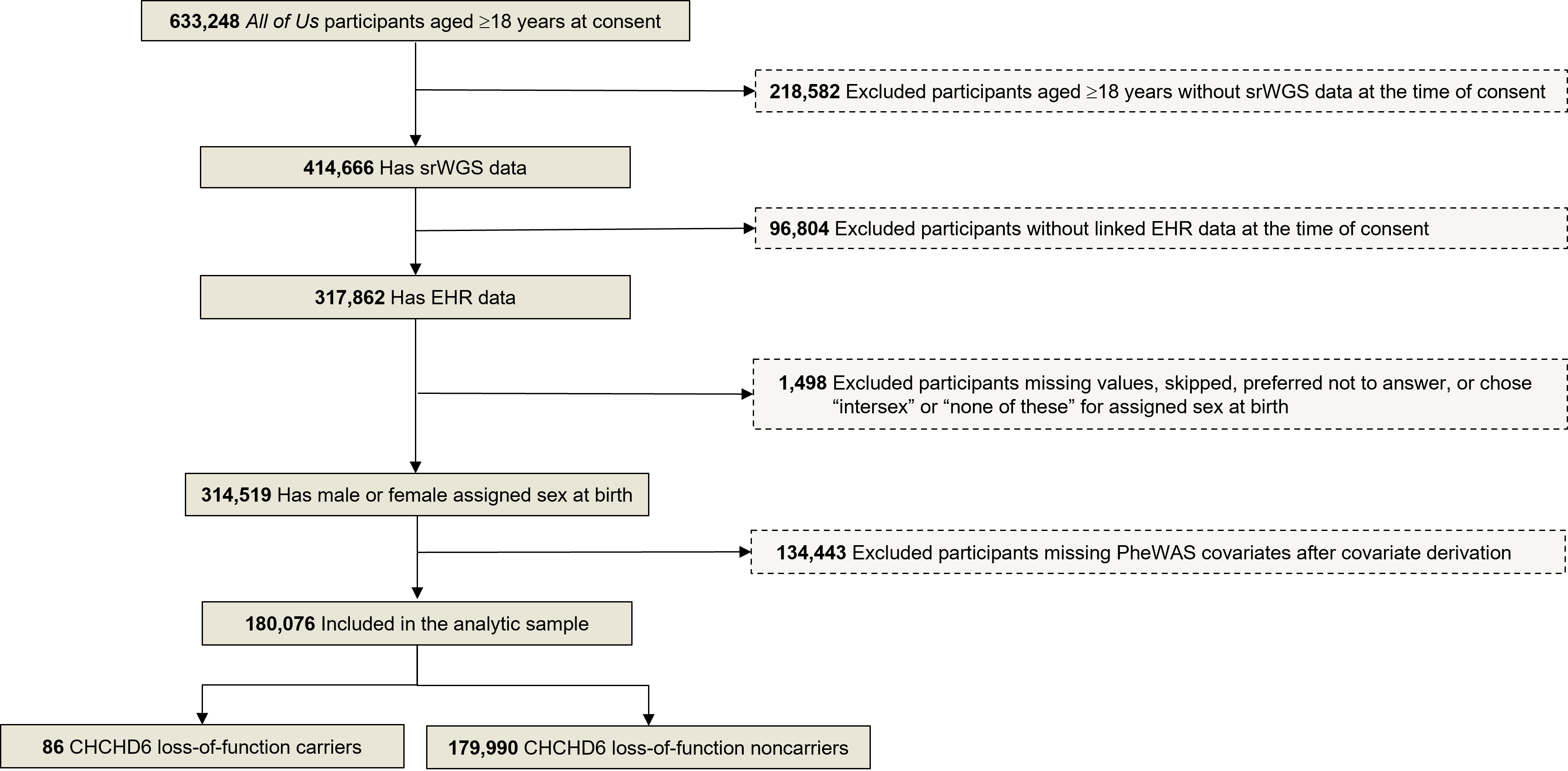
**

**Figure S1. Flowchart of CHCHD6 loss-of-function carrier cohort selection for covariate-adjusted PheWAS analysis from the *All of Us* Research Program.** Participants were selected from the *All of Us* Research Program using Controlled Tier Dataset version 8, including participants enrolled and consented through October 1, 2023. Among 633,248 participants aged ≥18 years at consent, participants without srWGS data, without linked EHR data, or with assigned sex at birth other than male or female or missing and declined responses were excluded. CHCHD6 rare predicted LoF carriers were defined using variants annotated as frameshift, splice-acceptor, splice-donor, or stop-gained, with allele frequency ≤0.001. This yielded an initial eligible case-control cohort of 314,519 participants, including 148 CHCHD6 LoF carriers and 314,371 noncarriers. After derivation of regression covariates and removal of participants with missing covariate data, the final covariate-complete analytic sample comprised 180,076 participants, including 86 CHCHD6 LoF carriers and 179,990 noncarriers. srWGS, short-read whole-genome sequencing; EHR, electronic health record; LoF, loss-of-function.

**
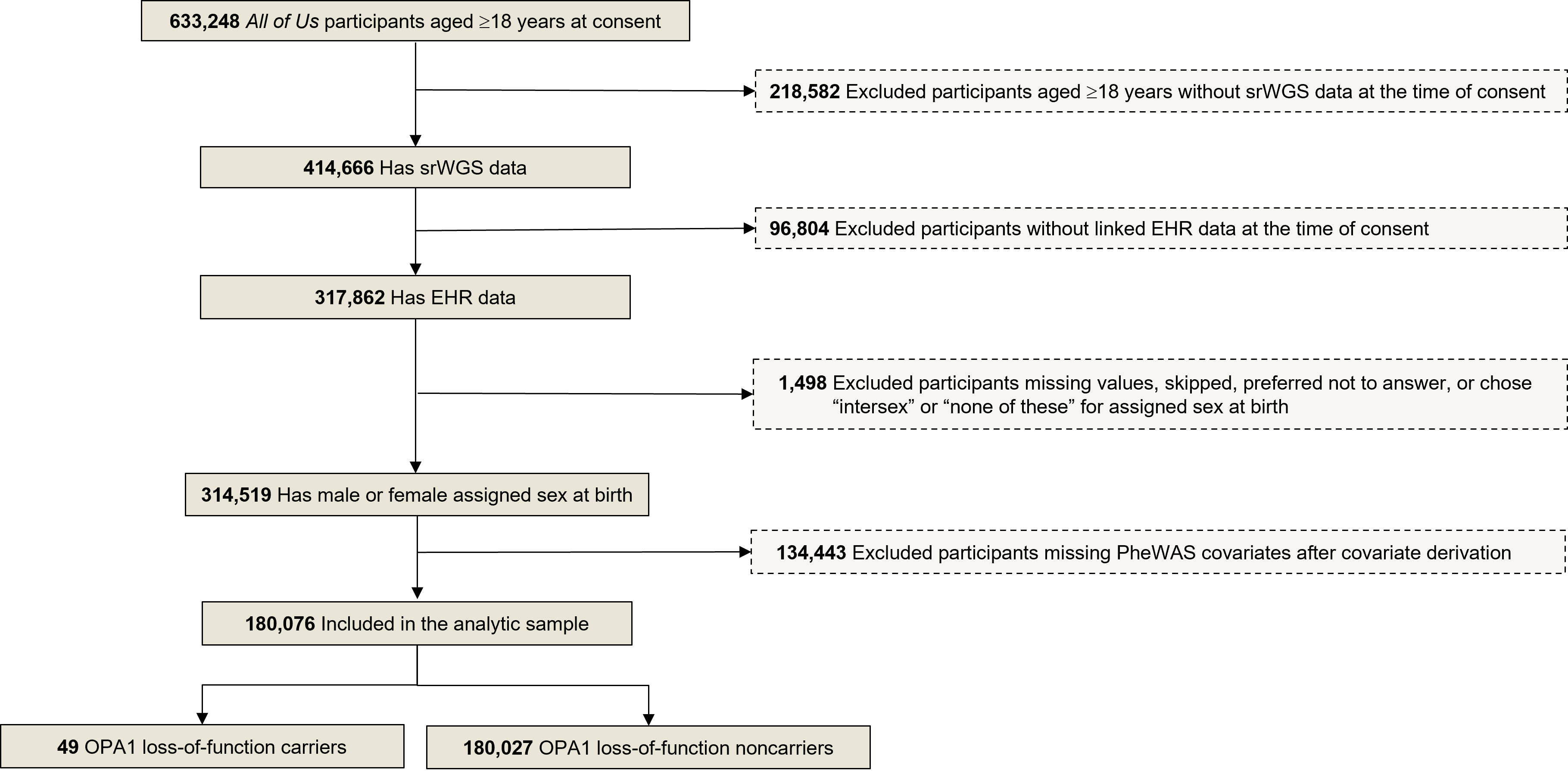
**

**Figure S2. Flowchart of OPA1 loss-of-function carrier cohort selection for covariate-adjusted PheWAS analysis from the *All of Us* Research Program.** Participants were selected from the *All of Us* Research Program using Controlled Tier Dataset version 8, including participants enrolled and consented through October 1, 2023. Among 633,248 participants aged ≥18 years at consent, participants without srWGS data, without linked EHR data, or with assigned sex at birth other than male or female or missing and declined responses were excluded. OPA1 rare predicted LoF carriers were defined using variants annotated as frameshift, splice-acceptor, splice-donor, or stop-gained, with allele frequency ≤0.001. This yielded an initial eligible case-control cohort of 314,519 participants, including 83 OPA1 LoF carriers and 314,436 noncarriers. After derivation of regression covariates and removal of participants with missing covariate data, the final covariate-complete analytic sample comprised 180,076 participants, including 49 OPA1 LoF carriers and 180,027 noncarriers. srWGS, short-read whole-genome sequencing; EHR, electronic health record; LoF, loss-of-function.
